# Adaptive conduction delays and phase locking in spiking Haken Lighthouse networks

**DOI:** 10.1007/s00422-026-01064-2

**Published:** 2026-09-21

**Authors:** Stephen Coombes, Rüdiger Thul, Stefan Ruschel, Rachel Nicks

**Affiliations:** School of Mathematical Sciences, University of Nottingham, Nottingham, NG7 2RD UK

**Keywords:** Haken Lighthouse model, Phase locking, Conduction delays, White matter plasticity

## Abstract

We develop a theory of phase-locked activity in delayed spiking networks using the Haken Lighthouse model as an analytically tractable event-based description of neural dynamics. For networks with fixed delays, we derive self-consistency conditions for phase-locked states and an associated linear stability theory formulated directly in terms of spike-time perturbations. The framework is illustrated for a delayed autapse, a reciprocally coupled two-cell network, and spatially structured rings with distance-dependent coupling and conduction delays, where circulant symmetry allows stability to be decomposed into Fourier modes. We then introduce an activity-dependent white matter plasticity rule in which myelination modulates axonal conduction speed and hence communication delay. This leads naturally to a slow–fast system with state-dependent delays, in which frozen phase-locked branches organise the adaptive dynamics. The plasticity rule selects commensurate delay–period relationships, providing a mechanism for the emergence of synchrony, other frequency-locked states, slow switching between competing phase-locked patterns, and the organisation of heterogeneous delays into discrete delay–period classes. Direct simulations of the event-driven network support the analytical predictions and illustrate how adaptive conduction can reshape the attractor structure of a delayed spiking network and generate long-timescale transitions. These results provide a tractable mathematical framework for studying how activity-dependent myelination may regulate temporal coordination, synchrony, and communication through coherence in spiking neural systems.

## Introduction

A central goal of theoretical neuroscience is to construct tractable models of spiking neuronal networks. Achieving this requires a single-node model capable of generating discrete events—action potentials—that, when embedded in a synaptic network, can give rise to the collective behaviours observed in biological neural systems. Simple spiking neural network models, such as those built from interacting integrate-and-fire units, exhibit rich emergent behaviours but remain notoriously difficult to analyse, especially when one moves beyond regular firing patterns or highly symmetric forms of coupling (Coombes et al. 2012). In contrast, firing-rate descriptions and coupled phase-oscillator models offer greater mathematical tractability, but necessarily average over, or otherwise suppress, some of the event-based structure of neural communication. The Haken Lighthouse model offers a promising bridge between these modelling paradigms. Introduced by Hermann Haken within the wider programme of Synergetics, it describes a network of pulse-coupled units whose firing is determined by the rotation of a phase-like variable driven by synaptic input (Haken 2002). It is therefore spiking, but in a form that preserves enough structure to make the analysis of collective states feasible.

A recent revisiting of the Lighthouse model developed new mathematical tools for analysing self-organisation in spiking networks, including synchrony, waves, bumps, and pattern-forming instabilities (Coombes 2025). That work also reaffirmed the value of the Lighthouse model as a link between spiking descriptions, phase-oscillator ideas, and rate-based neural fields. However, the treatment of delays in that study was limited. This is a significant restriction. Delays are not small technical corrections to neural dynamics: they are fundamental components of communication in nervous systems (Campbell 2007). Axonal propagation, synaptic processing, dendritic filtering, and long-range anatomical embedding all introduce timing constraints, and these timing constraints can shape synchrony, phase locking, travelling activity, multistability, and the response of a network to perturbations. Recent work on large-scale rate-based brain models has similarly shown that fixed white matter delays can strongly influence synchrony and collective dynamics across spatially distributed neural populations (Şayli and Coombes 2024).

Delays are also increasingly relevant for NeuroAI and neuromorphic computation. Spiking neural networks are attractive partly because their event-based nature suggests a route toward energy-efficient computation (Ornes 2025; Di Caterina et al. 2024). Yet event-based computation is intrinsically temporal. The timing of a spike, and not merely its occurrence, can carry information. In this setting, delays are not just imperfections to be engineered away; they are additional degrees of freedom that can enhance memory, temporal processing, and reservoir-like computation (Tavakoli and Longtin 2024). A mathematical theory of spiking networks that treats delays as primary dynamical ingredients is therefore needed both for neuroscience and for next-generation brain-inspired computing.

The biological motivation for treating delays seriously is strengthened by the growing evidence that white matter is plastic. Myelination controls axonal conduction speed and hence the delay with which signals arrive at their targets. It is now clear that myelination is activity dependent, with neural activity influencing oligodendrocyte lineage cells and modulating conduction properties through changes in myelin structure on a time-scale that is slow compared to neural dynamics (Gibson et al. 2014; Mount and Monje 2017; Xin and Chan 2020). From a dynamical perspective this means that delays should not always be viewed as fixed parameters. Instead, they may themselves evolve slowly in response to the activity that they help organise. Recent modelling work has begun to explore this idea in phase-oscillator and large-scale network settings, showing how adaptive myelination can promote synchronisation, resilience, and slow switching in delayed networks (Pajevic et al. 2014; Park and Lefebvre 2020; Talidou et al. 2022; Klinshov and Nekorkin 2024; Lefebvre et al. 2025; Pajevic et al. 2023; Ruschel et al. 2026; Jolly et al. 2026). The aim of the present paper is to develop an analogous theory directly at the level of a spiking Lighthouse network.

The main contribution of this paper is therefore twofold. First, we develop a framework for the existence and stability of phase-locked states in Haken Lighthouse networks with multiple fixed delays. The theory is formulated in terms of event times and leads naturally to a characteristic equation for perturbations of firing times. Secondly, we use this fixed-delay theory as the backbone for an adaptive model in which conduction speeds, and hence delays, evolve slowly according to a phenomenological rule for white matter plasticity. This produces a state-dependent delay problem in which the fast spiking dynamics and the slow evolution of conduction delays are coupled. The resulting framework gives a tractable way to study how adaptive delays can promote synchrony, organise switching between phase-locked states, and sculpt networks toward commensurate timing classes that preserve phase information along long-range pathways. In this sense, the model connects naturally with the communication-through-coherence viewpoint, in which the efficacy of interaction between neuronal populations depends on the phase at which inputs arrive (Fries 2005, 2015).

The structure of the paper is as follows. In Sect. 2 we introduce the Haken Lighthouse model on a graph, with synaptic interactions represented by causal kernels and communication delays. In Sect. 3 we derive the equations for phase-locked states with arbitrary network topology and multiple delays, and formulate their linear stability in terms of perturbations of firing times. Section 4 treats the single-node autapse as a minimal example in which folds of regular-spiking branches and dynamic instabilities of inter-spike intervals can be identified explicitly. In Sect. 5 we analyse a reciprocal two-node network, including synchronous, anti-synchronous, and asymmetric phase-locked states, and show how the two-delay problem can be reduced to an equivalent one-delay description. Section 6 treats ring networks with distance-dependent interactions and delays, where circulant structure allows the stability problem for twisted states to be diagonalised. In Sect. 7 we introduce a white-matter plasticity rule for the slow evolution of conduction speeds, develop a slow–fast interpretation of the adaptive system, and illustrate how the rule can promote convergence, switching, and commensurate timing. Finally, in Sect. 8 we summarise the main results and discuss how adaptive conduction delays in the Lighthouse model may provide a useful mathematical framework for studying biological white matter networks, and for understanding how delay-based mechanisms can regulate temporal coordination, synchrony and switching in spiking neural systems.

## The Lighthouse model

A recent revisiting of the Lighthouse model showed how its combination of event-driven spiking and synaptic filtering can be exploited to develop a mathematical theory of self-organised activity in spiking networks (Coombes 2025). In particular, it was shown that the model supports a range of collective states familiar from pattern-formation theory, including synchrony, travelling waves, localised bumps and instabilities of spatially homogeneous activity. These results position the Lighthouse model as a tractable framework for connecting pulse-coupled spiking dynamics with the geometric and spectral ideas more commonly used in the analysis of rate-based neural networks. The present paper builds on this viewpoint, but now places conduction delays at the centre of the analysis.

We consider the Haken Lighthouse model on a directed weighted graph with *N* nodes and connection strengths $w_{ij}\in \mathbb {R}$. The state of node *i* is described by a phase variable $\theta _i\in \mathbb {S}$, driven by a synaptic input $\psi _i\in \mathbb {R}$. The model takes the form

1 $$\begin{aligned} \begin{aligned} \frac{\textrm{d}}{\textrm{d}t}\theta _i(t)&= S\left( \psi _i(t)\right) ,\\ \psi _i(t)&= \sum _{j=1}^N w_{ij}\left( \eta _{ij}*s_j\right) (t),\\ s_j(t)&= \sum _{m\in \mathbb {Z}}\delta (t-T_j^m), \end{aligned} \end{aligned}$$

for i=1,…,N. Here, *S* is a non-negative firing-rate function, $\eta _{ij}$ is a causal synaptic response kernel from node *j* to node *i*, and ∗ denotes temporal convolution. The spike train $s_j$ is generated by the firing times $T_j^m$, of node *j* with $m\in \mathbb {Z}$. These firing times are determined by the threshold condition $\theta _j(T_j^m)=2\pi$, with the phase then understood to wrap around the circle. Thus the Lighthouse model is naturally formulated as an event-driven spiking system, in which the continuous evolution of the phase variables is coupled to the discrete sequence of spike times.

A typical choice for *S* is the sigmoidal function

2 $$\begin{aligned} S(x) = \frac{1}{1+\textrm{e}^{-\beta (x-h)}}, \end{aligned}$$

where $h\in \mathbb {R}$ is a threshold and β>0 controls the steepness. In what follows we shall assume more generally that *S* is positive, monotonically increasing, and at least once differentiable.

Communication delays enter through the synaptic kernels. In this paper we take $\eta _{ij}(t)=\eta (t-\tau _{ij})$, where $\tau _{ij}\ge 0$ is the propagation delay from node *j* to node *i*. For the underlying causal response we use the normalised α-function

3 $$\begin{aligned} \eta (t)=\alpha ^2t\textrm{e}^{-\alpha t}H(t), \end{aligned}$$

where *H* is the Heaviside function. The parameter α sets the synaptic time scale, with larger values corresponding to faster synaptic responses. Since η is the Green’s function of the linear differential operator

4 $$\begin{aligned} Q=\left( 1+\frac{1}{\alpha }\frac{\textrm{d}}{\textrm{d}t}\right) ^2, \end{aligned}$$

the synaptic input can equivalently be written in the filtered-spike form

5 $$\begin{aligned} Q\psi _i(t)=\sum _{j=1}^N w_{ij}s_j(t-\tau _{ij}). \end{aligned}$$

This representation makes explicit the mechanisms that will be central throughout the paper: spikes are communicated through delayed pathways, and the postsynaptic drive is obtained by filtering these delayed spike trains through a causal synaptic kernel.

## Phase-locked states

We now turn to the construction and stability of phase-locked states. The event-driven nature of the Lighthouse model makes it natural to formulate the problem directly in terms of firing times. Integrating (1) from one firing event at time $T_i^m$ to the next at time $T_i^{m+1}$ gives

6 $$\begin{aligned}&\theta _i(T_i^{m+1}) - \theta _i(T_i^{m}) = 2 \pi \nonumber \\&= \int _{0}^{T_i^{m+1}-T_i^m} \! \! \! \! \! \! \! \! \! \! \textrm{d}t \, S \left( \sum _{j=1}^N w_{ij} \! \! \! \! \! \! \! \! \! \! \sum _{\{ p \mid T_j^p < T_i^{m+1} \}} \! \! \! \! \! \! \! \! \! \! \eta (t +T_i^m -T_j^p - \tau _{ij}) \right) \end{aligned}$$

This expression is an exact firing-time map: the next spike of node *i* is determined by the delayed spikes from all presynaptic nodes that arrive before the threshold crossing.

We now define a phase-locked state according to $T_i^m = mT + \phi _i T$ with $\phi _i \in [0,1)$. In this case (6) reduces to the set of equations:

7 $$\begin{aligned} 2 \pi = \int _{0}^{T} \textrm{d}t \, S \left( \sum _{j=1}^N w_{ij} P(t +(\phi _i-\phi _j)T - \tau _{ij}) \right) , \end{aligned}$$

for i=1,…,N. Here, the *T*-periodic function *P*(*t*) is given by $P(t) = \sum _{m \in \mathbb {Z}} \eta (t - m T)$. This may be equivalently represented by a Fourier series of the form $P(t) = \sum _n P_n \textrm{e}^{\textrm{i}\omega _n t }$ with $\omega _n = 2 \pi n/T$

8 $$\begin{aligned} P_n = \frac{1}{T} \widehat{\eta }(\omega _n) , \quad \widehat{\eta }(\omega ) = \int _{-\infty }^ \infty \textrm{d}t \, \eta (t) \textrm{e}^{-\textrm{i}\omega t} . \end{aligned}$$

Here $\widehat{\eta }$ is recognised as the Fourier transform of η. For the α-function we have that

9 $$\begin{aligned} \widehat{\eta } (\omega ) = \frac{1}{(1+\textrm{i}\omega /\alpha )^2} . \end{aligned}$$

We note that we may also evaluate *P*(*t*) in closed form by recognising it as a geometric progression that may be summed to give

10 $$\begin{aligned} P(t)&= -\alpha ^2 \frac{\textrm{d}}{\textrm{d}\alpha } \sum _{m \le 0} \textrm{e}^{-\alpha (t-mT)} \nonumber \\&= \frac{\alpha ^2 \textrm{e}^{-\alpha t}}{1-\textrm{e}^{-\alpha T}}\left[ t + \frac{T \textrm{e}^{-\alpha T}}{1-\textrm{e}^{-\alpha T}} \right] , \qquad t \in [0, T). \end{aligned}$$

The system of equations (7) is invariant under the shift $\phi _i \rightarrow \phi _i + a$ for some arbitrary constant *a*. This highlights a phase-shift symmetry of the system, arising from the fact that the equations defining a phase-locked state only depend upon phase differences. The same system of equations is also invariant to a constant shift for one (and only one) of the delays, namely $\tau _{ij} \rightarrow \tau _{ij} + a$ for some pair (*i*, *j*). The same is true if a constant is added to *all* delays. This arises because the integral of a periodic function over its period is invariant with respect to the choice of an origin. A consequence of this is that if there is only one delay in the system then the emergent period is independent of that delay. We also note that if *S* is a linear function then (7) is independent of all delays. If we fix one phase, say $\phi _1=0$, then (7) gives a system of *N* equations in the *N* unknowns $(T, \phi _2, \ldots , \phi _N)$ (for fixed delays). Moreover, if it proves convenient we might fix one delay in the system and refer all others to this.

To determine the linear stability of this phase-locked state we consider a perturbed flow denoted by $\widetilde{\theta }_i(t) = {\theta }_i(t) + \delta {\theta }_i(t)$ with a corresponding set of perturbed firing times that we denote as $\widetilde{T}_i^m =T_i^m + \delta \widetilde{T}_i^m$. This perturbed system can be integrated to yield (6) under the replacement $\theta _i \rightarrow \widetilde{\theta }_i$ and $T_i^m \rightarrow \widetilde{T}_i^m$. A Taylor expansion of this set of equations for small perturbations yields

11 $$\begin{aligned} 0&\simeq \int _0^T \textrm{d}t \, S' \left( \sum _{j=1}^N w_{ij} P(t+ A_{ij}) \right) \times \nonumber \\&\sum _{k=1}^N w_{ik} \sum _{p \in \mathbb {Z}} \eta ' (t+ pT + A_{ik}) \left( \delta T_i^m - \delta T_k^{m-p} \right) \nonumber \\&+ ( \delta T_i^{m+1} - \delta T_i^{m} ) S \left( \sum _{j=1}^N w_{ij} P(A_{ij}) \right) , \end{aligned}$$

where $A_{ij} = (\phi _i-\phi _j)T -\tau _{ij}$. This linear difference equation has solutions of the form $\delta T_i^m = \delta T_i \textrm{e}^{m \lambda }$ with $\lambda \in \mathbb {C}$. These solutions satisfy the system of linear equations

12 $$\begin{aligned} \left( \textrm{e}^{\lambda } - 1 \right) \dot{\theta }_i \delta T_i + \sum _{j=1}^N \mathcal {L}_{ij} (\lambda ) \delta T_j = 0, \end{aligned}$$

where $$\dot{\theta }_i = S (\sum _j w_{ij} P(A_{ij}))$$, $$\mathcal {L}_{ij}(\lambda ) = -w_{ij}(\lambda ) + \delta _{ij} \sum _k w_{ik}(0)$$, $$w_{ij} (\lambda ) = w_{ij} G_{ij} (\lambda )$$ and

13 $$\begin{aligned} G_{ik}(\lambda )&= \sum _{p \in \mathbb {Z}} \textrm{e}^{-p \lambda } \int _0^T \textrm{d}t \, S' \left( \sum _{j=1}^N w_{ij} P(t+A_{ij}) \right) \nonumber \\&\quad \times \eta '(t+pT + A_{ik}) . \end{aligned}$$

Introducing the N×N diagonal matrix $\dot{\Theta }$ with components $[\dot{\Theta }]_{ij} = \dot{\theta }_i \delta _{ij}$ means that we can write (12) in the more compact form $\mathcal {M}(\lambda ) \delta T = 0$, where $\delta T = (\delta T_1, \ldots , \delta T_N)$ and

14 $$\begin{aligned} \mathcal {M}(\lambda ) = \left( \textrm{e}^{\lambda } - 1 \right) \dot{\Theta } + \mathcal {L} (\lambda ). \end{aligned}$$

For non-trivial solutions we must have that $\mathcal {E}(\lambda ) = 0$ where $\mathcal {E}(\lambda ) = \det \mathcal {M}(\lambda )$. A phase-locked state will be stable if $\text {Re} \, \lambda <0$. We note that (1,1,…,1) is an eigenvector of $\mathcal {L}(0)$ with eigenvalue zero. Hence, $\mathcal {E}(0) = 0$. This is expected since the system has phase shift symmetry.

A computationally useful form for $$G_{ij}(\lambda )$$ can be obtained by using a Fourier integral representation of $\eta^{\prime }$ as:

15 $$\begin{aligned} \eta '(t) = \frac{1}{2 \pi } \int _{-\infty }^\infty \textrm{d}k \, (\textrm{i}k) \widehat{\eta } (k) \textrm{e}^{\textrm{i}k t} . \end{aligned}$$

Using this we find

16 $$\begin{aligned} G_{ij}(\lambda ) = \frac{1}{T} \sum _{n\in \mathbb {Z}} f_{i} (\widetilde{\omega }_n (\lambda )) \, \widehat{\eta } (\widetilde{\omega }_n (\lambda )) \textrm{e}^{\textrm{i}(\widetilde{\omega }_n (\lambda )) A_{ij}} , \end{aligned}$$

where $$\widetilde{\omega }_n (\lambda ) = \omega _n -\textrm{i}\lambda /T$$ ($$\omega _n = 2 \pi n /T$$) and

17 $$\begin{aligned} f_i(\omega ) = \int _0^T \textrm{d}t \, S' ( X_i(t)) (\textrm{i}\omega )\textrm{e}^{\textrm{i}\omega t} . \end{aligned}$$

where $X_i(t) = \sum _{j=1}^N w_{ij} P(t+A_{ij})$, and we have made use of the Dirac comb identity $2 \pi \sum _{n \in \mathbb {Z}} \delta (x-2 \pi n) = \sum _{p \in \mathbb {Z}} \textrm{e}^{\textrm{i}p x}$. By writing $Y_i(t)=S'(X_i(t))$ as a Fourier series with Fourier coefficients $Y_{i,n} \in \mathbb {C}$ we may evaluate (17) to the computationally useful form $f_i(\omega _n) = \textrm{i}\omega _n T \, Y_{i,-n}$.

In practice, the spectral equation $\mathcal {E}(\lambda )=0$ can be evaluated as follows. For a given phase-locked state, the period *T* and phases $\phi _i$ are first determined from (7). For each trial value of λ, the quantities $G_{ij}(\lambda )$ are then computed from the Fourier representation (16), with the infinite sum over *n* truncated symmetrically to $|n|\le n_{\max }$. This truncation is used only to evaluate the entries of the N×N matrix $\mathcal {M}(\lambda )$; it does not enlarge the dimension of the stability matrix. The roots of the scalar nonlinear equation $\mathcal {E}(\lambda )=\det \mathcal {M}(\lambda )=0$ are then found directly, or in special cases to determine potential bifurcation points by solving the real and imaginary parts of $\mathcal {E}(\textrm{i}\omega )=0$. Thus the Fourier truncation provides a spectral approximation of the delayed synaptic response, whereas the determinant is always taken over the network degrees of freedom.

In the examples below, this general construction will be specialised first to a single neuron with delayed self-feedback, then to a reciprocal two-node network, and finally to circulant ring networks where the spatial symmetry allows further diagonalisation.

## A single node with an autapse

We first apply the phase-locking framework to the simplest possible delayed network: a single Haken neuron with recurrent self-feedback. This example is useful for two reasons. First, it provides a minimal setting in which the role of delay can be isolated and understood. Second, it illustrates how the general firing-time stability theory detects both folds of regular-spiking branches and dynamic instabilities associated with modulations of the inter-spike intervals.

We consider a single neuron with two forms of self-coupling: an instantaneous autapse (self synapse) of strength $w_0$ and a delayed autapse of strength $w_\tau$ and delay τ. The instantaneous term breaks the delay-shift invariance discussed in the previous section, allowing the period of a regular spike train to depend non-trivially on the delay. Previous work on (perfect) integrate-and-fire and Hodgkin-Huxley-type neuron models, whose recurrent inputs are delayed versions of their output spike trains, has shown that multi-stability of periodic spike trains is possible (Foss et al. 1996). Other work by Klinshov et al. has shown that simple phase oscillator and Hodgkin-Huxley models with pulsatile delayed feedback can also exhibit so-called “jittering", whereby a periodic spike train destabilises to temporal patterns with non-equal interspike intervals (Klinshov et al. 2015). The Haken autapse displays analogous behaviour, but here the bifurcation structure can be obtained directly from the event-time formulation.

Following our earlier notation and dropping node labels (as we only consider one Haken neuron here), we write the *m*th inter-spike interval (ISI) as $\Delta ^{m} = T^{m+1}-T^m$. For a periodic spike train we have simply that $\Delta ^m = T$ for all *m*. Denoting the self-delay by τ and the self-connection strength by $w_\tau$, the period T=T(τ) is then implicitly defined from equation (7) as the solution to $\mathcal {H}(T,\tau ) = 0$, where

18 $$\begin{aligned} \mathcal {H}(T,\tau ) = \int _{0}^{T} \textrm{d}t \, S \left( w_0 P(t) + w_\tau P(t - \tau ) \right) - 2 \pi . \end{aligned}$$

From the linear stability analysis centred around equation (14), the periodic spike-train will be stable if $\text {Re} \, \lambda <0$ where $(\textrm{e}^{\lambda } - 1)\dot{\theta } - [\mathcal {G}(\lambda ) - \mathcal {G}(0)]=0 \equiv \mathcal {E}(\lambda )$, where

19 $$\begin{aligned} \mathcal {G}(\lambda )&= \sum _{p \in \mathbb {Z}} \textrm{e}^{-p \lambda } \int _0^T \textrm{d}t \, S' \left( w_0 P(t) + w_\tau P(t - \tau ) \right) \nonumber \\&\quad \times \left[ w_0 \eta '(t+pT) + w_\tau \eta '(t+pT-\tau ) \right] . \end{aligned}$$

Here, $\dot{\theta } = S(w_0 P(0)+ w_\tau P(-\tau ))$. Expanding the characteristic equation near λ=0 shows that a double root occurs when $\mathcal {E}(0)=0=\mathcal {E}'(0)$, giving $\dot{\theta }-\mathcal {G}'(0)=0$. We now show how this condition is related to a fold of the periodic spike-train branch defined by $\mathcal {H}(T,\tau )=0$. Differentiating (18) with respect to *T*, with τ held fixed, gives

20 $$\begin{aligned} \frac{\partial \mathcal {H}}{\partial T} = \dot{\theta } + \int _0^T \textrm{d}t \, S'(X)\frac{\partial X}{\partial T}, \end{aligned}$$

where $$X=w_0P(t)+w_\tau P(t-\tau )$$. Since

21 $$\begin{aligned} \frac{\partial X}{\partial T} = \sum _{p\in \mathbb {Z}} p \left[ w_0\eta '(t+pT)+w_\tau \eta '(t+pT-\tau )\right] , \end{aligned}$$

and $\mathcal {G}'(0)=-\int _0^T \textrm{d}t \,S'(X)\partial X/\partial T$, it follows that $\partial \mathcal {H}/\partial T=\dot{\theta }-\mathcal {G}'(0)=\mathcal {E}'(0)$.

To relate this condition to the geometry of the solution branch, implicit differentiation of $\mathcal {H}(T,\tau )=0$ gives

22 $$\begin{aligned} \frac{\textrm{d}\tau }{\textrm{d}T} = -\frac{\partial \mathcal {H}/\partial T}{\partial \mathcal {H}/\partial \tau }. \end{aligned}$$

Hence, provided that $\partial \mathcal {H}/\partial \tau \ne 0$, the condition $\partial \mathcal {H}/\partial T=0$ corresponds to $\textrm{d}\tau /\textrm{d}T=0$, or equivalently to a vertical tangent when the branch is plotted as T=T(τ). Thus a fold of the periodic spike-train branch coincides with the double-zero condition $\mathcal {E}(0)=0=\mathcal {E}'(0)$. A nondegenerate fold additionally requires $\partial ^2\mathcal {H}/\partial T^2\ne 0$.

For $\lambda = \textrm{i}\omega$ a dynamic instability (signalling the onset of an oscillatory modulation of the ISIs) can occur at some value of τ, and this is defined by the simultaneous solution of

23 $$\begin{aligned} \begin{aligned} \dot{\theta } \left( \cos \omega -1 \right)&= \left( \text {Re} \, \mathcal {G}(\textrm{i}\omega ) - \mathcal {G}(0) \right) ,\\ \dot{\theta } \sin \omega&= \text {Im} \, \mathcal {G}(\textrm{i}\omega ) , \end{aligned} \end{aligned}$$

for the pair $(\tau , \omega )$. The frequency ω characterises modulation with respect to the discrete spike index *m*, rather than physical time. A perturbation proportional to $\textrm{e}^{\textrm{i}m\omega }$ repeats after *q* spikes when

24 $$\begin{aligned} \frac{\omega }{2\pi }=\frac{p}{q}, \qquad p,q\in \mathbb {Z}. \end{aligned}$$

Since successive spikes of the underlying periodic train are separated by *T*, a cycle of the modulation then spans a physical time *qT*, and the corresponding physical angular frequency is ω/T. In particular, $\omega =\pi$ corresponds to a period-two modulation with alternating long and short ISIs, while $\omega =2\pi /3$ gives a repeating sequence of three ISIs. If $\omega /(2\pi )$ is irrational, the modulation does not repeat after a finite number of spikes.

Thus the onset of instability is naturally associated with “jittering” of the spike train. Close to the instability, the ISIs have the leading-order form

25 $$\begin{aligned} \Delta ^m=T+\varepsilon \cos (m\omega +\phi _0)+O(\varepsilon ^2), \end{aligned}$$

where $\varepsilon \ll 1$ is the amplitude of the ISI modulation and $\phi _0$ is an arbitrary phase determined by the initial perturbation.

**Fig. 1:**
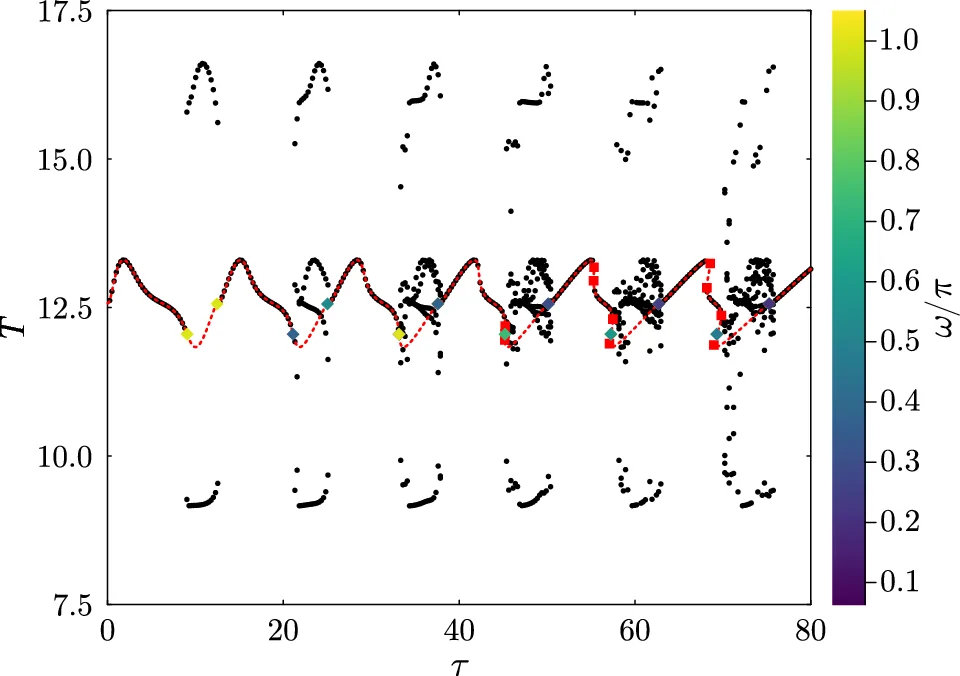
Bifurcation structure of the Haken lighthouse autapse model in the (τ,T) plane, combining semi-analytical branch information with direct numerical simulations. The dotted red curves show regular-spiking solution branches defined implicitly by $\mathcal {H}(T,\tau )=0$. Small red squares mark fold points obtained from direct solution of the double zero condition. Coloured diamond markers indicate dynamic-instability points obtained from direct solution of (23), with marker colour representing $\omega /\pi$. Black dots show late-time ISIs obtained from direct delayed simulations of the autapse model after discarding transients. Parameter values are $w_{0}=1$, $w_{\tau }=-1$, α=1, β=10, h=0

The resulting bifurcation structure is shown in Fig. 1. The red dotted curves are the regular-spiking branches obtained by solving $\mathcal {H}(T,\tau )=0$. Folds of these branches agree with the double-zero condition $\mathcal {E}'(0)=0$, shown by red square markers. Dynamic instabilities obtained from (23) are shown by coloured diamond markers, with the colour encoding the modulation frequency $\omega /\pi$. Direct numerical simulations of the delayed autapse model are superimposed as black points, showing the late-time ISIs after transients have been discarded. Along stable portions of the regular-spiking branches the simulations collapse onto a single ISI value. Beyond dynamic-instability points, the single ISI splits into multiple values, indicating jittering or higher-period ISI patterns. Thus the figure illustrates both the multistability of regular spike trains and the loss of regular spiking through dynamic modulation of the ISIs.

Related branch structures have been analysed in detail for a theta neuron with delayed pulsatile self-feedback (Laing and Krauskopf 2022). In that setting, the explicit solvability of the theta-neuron dynamics with impulsive feedback permits analytical expressions for the existence and stability of periodic solutions, including repeated branches, delay-induced multistability and their saddle-node bifurcations. These results establish that the proliferation of periodic spike trains with increasing delay is not specific to the Lighthouse model. The role of the present autapse example is instead to show how analogous branch structure, together with dynamic instabilities producing non-uniform inter-spike intervals, is recovered from the general firing-time formalism developed in Sect. 3 for filtered synaptic interactions.

## Phase-locked states in a reciprocal two-node network

We next consider a reciprocal two-node network. This is the smallest network in which one can distinguish synchronous, anti-synchronous and asymmetric phase-locked states. It therefore provides a useful bridge between the autapse calculation and larger networks with non-trivial phase structure. We take N=2, with symmetric coupling and a common delay, so that $w_{ij}=\delta _{ij}+(1-\delta _{ij})w$ and $\tau _{ij}=(1-\delta _{ij})\tau$. Introducing the phase difference $\phi =\phi _2-\phi _1$, the phase-locking equations (7) reduce to a pair of equations for (T,ϕ) given by $\mathcal {H}(T,\pm \phi )=0$. Here $\mathcal {H}$ is 1-periodic in its second argument and given by

26 $$\begin{aligned} \mathcal {H}(T,\phi ) = \int _0^T \textrm{d}t \, S \left( P(t) + w P(t + \phi T -\tau ) \right) -2 \pi . \end{aligned}$$

From the form of (26) we see that if there is a solution for a given value of τ the same solution is generated under the replacement $\tau \rightarrow \tau + kT$ for $k \in \mathbb {Z}$. We note that a synchronous solution with ϕ=0 satisfies $\mathcal {H}(T,0)=0$ and an anti-synchronous solution satisfies $\mathcal {H}(T,1/2)=0$, namely each solution is specified by only one equation that we write in the form $\mathcal {H}(T,\psi )=0$ for $\psi \in \{0,1/2 \}$. For these cases we may construct the matrix w(λ) as

27 $$\begin{aligned} w_{ij}(\lambda ) = \frac{1}{T} \sum _{n\in \mathbb {Z}} f(\widetilde{\omega }_n (\lambda )) \, \widehat{\eta } (\widetilde{\omega }_n (\lambda )) B_{ij} (\widetilde{\omega }_n (\lambda )) , \end{aligned}$$

where

28 $$\begin{aligned} B(\omega ) = \begin{bmatrix} 1 & w \textrm{e}^{\textrm{i}\omega [\psi T-\tau ]} \\ w \textrm{e}^{\textrm{i}\omega [\psi T-\tau ]} & 1 \end{bmatrix} , \end{aligned}$$

and

29 $$\begin{aligned} f(\omega ) = \int _0^T \textrm{d}t \, S' \left( P(t) + w P(t+ \psi T - \tau ) \right) (\textrm{i}\omega )\textrm{e}^{\textrm{i}\omega t} . \end{aligned}$$

The matrix *B* has eigenvalues $\gamma _\pm (\omega ) = 1 \pm w e^{\textrm{i}\omega [\psi T-\tau ]}$ whose eigenvectors (1, 1) and (1,-1) are independent of ω. Thus we may diagonalise the linearised system (12) by considering perturbations δT parallel to these directions. The synchronous and anti-synchronous solutions are stable if $\text {Re} \, \lambda <0$ where $\mathcal {E}_\pm (\lambda ) = 0$ and

30 $$\begin{aligned} \mathcal {E}_\pm (\lambda ) = \left( \textrm{e}^\lambda -1 \right) \dot{\theta } + \kappa - \Gamma _{\pm }(\lambda ) . \end{aligned}$$

Here $$\dot{\theta } = S(P(0) + w P(\psi T - \tau ) )$$ and

31 $$\begin{aligned} \Gamma _{\pm }(\lambda )&= \frac{1}{T} \sum _{n\in \mathbb {Z}} f(\widetilde{\omega }_n (\lambda )) \, \widehat{\eta } (\widetilde{\omega }_n (\lambda )) \gamma _\pm (\widetilde{\omega }_n (\lambda )) ,\end{aligned}$$

32 $$\begin{aligned} \kappa&= \frac{1}{T} \sum _{n \in \mathbb {Z}} f(\omega _n ) \, \widehat{\eta } (\omega _n) \left[ 1+ w \textrm{e}^{\textrm{i}\omega _n [\psi T-\tau ]}\right] . \end{aligned}$$

We now restrict attention to $\lambda \in \mathbb {R}$. In this case $\mathcal {E}_\pm (\lambda ) \in \mathbb {R}$ and we note the following: $\mathcal {E}_+ (0) = 0$, $\mathcal {E}_- (0) = 2 R$, where

33 $$\begin{aligned} R&= \frac{w}{T} \sum _{n \in \mathbb {Z}} f(\omega _n ) \, \widehat{\eta } (\omega _n) \textrm{e}^{\textrm{i}\omega _n (\psi T-\tau )} \nonumber \\&= w \int _0^T \textrm{d}t \, S'(P(t) + w P(t+\psi T - \tau ) ) P'(t +\psi T -\tau ) \nonumber \\&= \frac{1}{T} \frac{\partial }{\partial \psi } \mathcal {H}(T,\psi ) . \end{aligned}$$

Assuming that *f* and $\widehat{\eta }$ are decaying functions of their arguments then we have that, for $T \le 2 \tau$, $\kappa \ge \Gamma _{\pm }(\lambda )$ so that $\lim _{\lambda \rightarrow \infty } \mathcal {E}_{\pm }(\lambda ) = \infty$. For R<0 the graph of $\mathcal {E}_-(\lambda )$ must cross the zero axis at a positive value of λ. Hence, a solution (synchronous or anti-synchronous) is linearly unstable if R<0. A double zero occurs at R=0 indicating a bifurcation at this point. It is also possible to relate the real-eigenvalue stability condition to the geometry of the solution branches T=T(τ). Since ψ and τ enter $\mathcal {H}$ through the combination $\psi T-\tau$, equation (33) gives $\partial \mathcal {H}/\partial \tau =-R$. Implicit differentiation of $\mathcal {H}(T,\psi )=0$ with respect to τ, with ψ fixed, therefore gives

34 $$\begin{aligned} \frac{\textrm{d}T}{\textrm{d}\tau } =-\frac{\partial \mathcal {H}/\partial \tau }{\partial \mathcal {H}/\partial T} =\frac{R}{\partial \mathcal {H}/\partial T}. \end{aligned}$$

Provided that $\partial \mathcal {H}/\partial T\ne 0$, the condition R=0 gives $\textrm{d}T/\textrm{d}\tau =0$. It therefore corresponds to a stationary point of T=T(τ) and to the symmetry-breaking bifurcation at which an asymmetric branch attaches to a synchronous or anti-synchronous branch. This should be distinguished from a saddle-node fold, which occurs when $\partial \mathcal {H}/\partial T=0$ while R≠0. At such a point $\textrm{d}T/\textrm{d}\tau$ becomes unbounded, corresponding to a vertical tangent of the branch.

For phase-locked states that are neither synchronous nor anti-synchronous, the two existence equations are $\mathcal {H}(T,\phi )=0$ and $\mathcal {H}(T,-\phi )=0$. When T=2τ, these equations are invariant under the reflection $\phi \mapsto 1-\phi$. Consequently, asymmetric solutions occur in conjugate pairs: if $\overline{\phi }\in (0,1/2)$ is a solution, then there is a corresponding solution with phase difference $1-\overline{\phi }\in (1/2,1)$. It is therefore sufficient to determine the member $\overline{\phi }\in (0,1/2)$ of each pair. From the periodicity of the system, the corresponding branches in the (τ,T) plane can be continued according to τ=(p+1/2)T, for p=0,1,2,…. A second family is obtained when τ=T. In this case, if $\overline{\phi }\in (0,1/2)$ satisfies $\mathcal {H}(T,\overline{\phi })=0$ with T=τ, then its conjugate solution has phase difference $1-\overline{\phi }\in (1/2,1)$. Periodicity generates the associated asymmetric branches according to τ=pT, for p=1,2,3,…. The stability of these asymmetric branches can be determined using the general machinery developed around equation (14).

We expect asymmetric branches to connect maxima and minima of symmetric branches (where stability changes) in a pitchfork bifurcation. A proof is given in Appendix A.

**Fig. 2:**
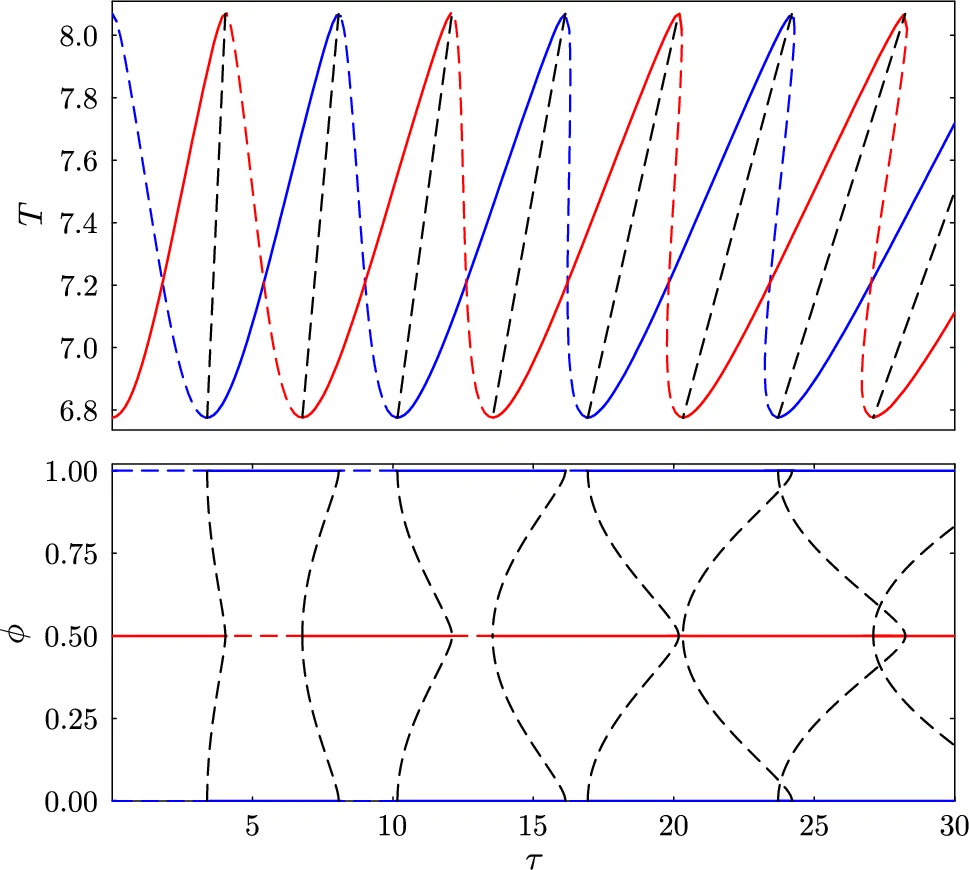
Bifurcation diagram for a reciprocal two-node Haken network with a common delay τ. The upper panel shows the period T=T(τ), and the lower panel shows the corresponding phase difference $\phi =\phi (\tau )$. Solid curves indicate stable phase-locked states and dashed curves indicate unstable states. The branches with ϕ=0 modulo 1 correspond to synchrony, while branches with ϕ=1/2 correspond to anti-synchrony. Additional asymmetric branches occur in phase-conjugate pairs related by $\phi \mapsto 1-\phi$ and attach to extrema of the symmetric branches. Parameters are α=1, w=1, β=10 and h=0

The resulting bifurcation structure is shown in Fig. 2. The upper panel shows the period *T* as a function of the common delay τ, while the lower panel shows the corresponding phase difference ϕ. Solid curves denote stable phase-locked states and dashed curves denote unstable states. The synchronous branches have ϕ=0 modulo 1, whereas the anti-synchronous branches have ϕ=1/2. The asymmetric branches appear in pairs related by the transformation $\phi \mapsto 1-\phi$ and connect neighbouring extrema of the symmetric branches. Thus even in the reciprocal two-node network the delayed Lighthouse model supports multistability between synchronous and anti-synchronous phase-locked states (organised around unstable asymmetric ones).

Related delay-dependent phase-locking structures have previously been obtained in other spiking and pulse-coupled oscillator models. Coombes and Lord studied periodic firing and phase selection in a pair of pulse-coupled integrate-and-fire neurons with axonal, synaptic and dendritic delays, constructing the associated bifurcation and stability diagrams (Coombes and Lord 1997). Klinshov and Nekorkin subsequently showed that a pair of time-delayed pulse-coupled oscillators similarly possess repeated synchronisation regions, with in-phase and anti-phase regimes alternating as the delay is increased (Klinshov and Nekorkin 2011). More recently, explicit existence and stability conditions for synchronous and alternating periodic solutions have been derived for a pair of delay-coupled active theta neurons (Laing 2025). In the latter case, the symmetric branches are connected by unstable symmetry-broken solutions arising at extrema of the period as a function of delay.

The repeated synchronous and anti-synchronous branches in Fig. 2, together with the asymmetric branches connecting their extrema, should therefore be viewed as Lighthouse-model manifestations of a bifurcation structure already observed in other delayed spiking and pulse-oscillator descriptions. The purpose of the reciprocal pair here is to demonstrate how this structure is recovered from the general firing-time framework of Sect. 3 and to provide the frozen-delay backbone for the adaptive dynamics studied later in Sect. 7.2.

### From two delays to one

For a two-node network we may also consider two distinct delays, with $\tau _{12}=\tau _1$ and $\tau _{21}=\tau _2$. In equation (26) the dependence of $\mathcal {H}$ on the common delay τ was suppressed in the notation. We now make this dependence explicit by writing

35 $$\begin{aligned} \mathcal {H}(T,\phi ;\tau ) = \int _0^T \textrm{d}t\,S\left( P(t)+wP(t+\phi T-\tau )\right) -2\pi . \end{aligned}$$

The defining equations for a phase-locked state with two distinct delays are therefore $\mathcal {H}(T,\phi ;\tau _1)=0$ and $\mathcal {H}(T,-\phi ;\tau _2)$ =0.

Noting that the arguments to the integrands are $\phi T -\tau _1$ and $-\phi T-\tau _2$ it is natural to consider a new notion of phase and delay according to $\phi T -\tau _1 = \chi T - \overline{\tau }$ and $-\phi T -\tau _2 = -\chi T - \overline{\tau }$. Solving this pair of equations gives $\overline{\tau } = (\tau _1+\tau _2)/2$ and $\chi = \phi -\delta /T$ where $\delta = (\tau _1-\tau _2)/2$. We may then write $\mathcal {H}(T,\phi ;\tau _1) = \mathcal {H}(T,\chi ;\overline{\tau })$ and $\mathcal {H}(T,-\phi ;\tau _2) = \mathcal {H}(T,-\chi ;\overline{\tau })$. Thus, we are led to an equivalent problem with only a single effective mean delay that we must solve for $(\chi ,T) = (\chi (\overline{\tau }),T(\overline{\tau }))$. This can be done using the prescription above (for a single delay), from which we may construct $(\tau _1, \tau _2, T, \phi )$ parametrically in terms of $(\overline{\tau },\delta )$ as $(\overline{\tau } + \delta , \overline{\tau } - \delta , T(\overline{\tau }), \chi (\overline{\tau }) + \delta /T(\overline{\tau }) \! \mod 1)$. Consequently, unequal reciprocal delays do not introduce a new existence problem for phase-locked states in the two-node network: they shift the observed phase difference relative to the effective common-delay solution. The case of two delays is taken up again later in Sect. 7.2.

## Phase-locked states in a ring with space dependent delays

Here we consider a Lighthouse network with interactions whose strength and delay both depend on distance. For simplicity we consider nodes arranged on a ring with the distance between nodes *i* and *j* given by $\operatorname {dist} (i,j) = \min (|i-j|, N-|i-j|)d$, for some spatial scale set by *d*. The space-time connectome then has a circulant matrix representation with $w_{ij} = w_{\operatorname {dist} (i,j)}$ and $\tau _{ij} = \tau _{\operatorname {dist} (i,j)}$ with rows generated by $w_{n} = w(\operatorname {dist} (0,n))$ for n=0,…,N-1 for some function *w* and delays chosen as $\tau _n = \operatorname {dist} (0,n)/v$. Here v>0 represents a common conduction speed. From the form of (7) we can expect the emergence of *twisted* states defined by $\phi _i = i q /N$, so that $\phi _i - \phi _j = (i-j)q/N$ $(\! \! \! \! \mod 1)$ for some $q \in \mathbb {Z}$. For q=0 this would represent the synchronous state and for q=1 the so-called splay state. We note that mode *q* and N-q are conjugate (reversed) twisted states. For a fixed value of *q* the emergent period *T* is given implicitly from (7) as the solution to $\mathcal {H}_q(T)=0$, where

36 $$\begin{aligned} \mathcal {H}_q(T) = \int _{0}^{T} \textrm{d}t \, S \left( \sum _{n=0}^{N-1} w_{n} P(t + A_{n,q}) \right) -2 \pi , \end{aligned}$$

and $A_{n,q} = n q T/N - \tau _{n}$ with $w_n=w_{N-n}$ and $\tau _n=\tau _{N-n}$ (reflecting the fact that both coupling strength and delay depend only on ring distance). The circulant properties of the space-time connectome mean that the matrix $\mathcal {L}(\lambda )$ is also circulant. Specifically, the matrix G(λ) has a row generator given by (16) under the replacement ij→n and $f_i (\omega ) \rightarrow f(\omega ) = \int _0^T \textrm{d}t \, S'(X_q(t)) (\textrm{i}\omega ) \textrm{e}^{\textrm{i}\omega t}$ for n=0,…,N-1 and $X_q(t) = \sum _{n=0}^{N-1} w_n P(t+A_{n,q})$. Similarly we will denote the row generators for $w_{ij}(\lambda )$ using the notation $w_n(\lambda )$.

In general for $\lambda \ne 0$ the matrices w(λ) and *w*(0) defined after equation (12) will not have a common eigenspace. However, when these are circulant they do and we may exploit this to diagonalise (12). After introducing right (left) normalised eigenvectors of *w*(0) as $r^\mu$ ($l^\mu$), we can write $w(\lambda ) = \sum _{\mu } \gamma _\mu (\lambda ) l^\mu \otimes r^\mu$, where ⊗ denotes the tensor product. Here, $\gamma _\mu (\lambda )$ can be constructed by projection of w(λ) on to $l^\mu$ and $r^\mu$ as $\gamma _\mu (\lambda ) = \left( l^\mu \right) ^\top w(\lambda ) r^{\mu } = \sum _{i,j} l^\mu _i w_{ij}(\lambda ) r^\mu _j$. By introducing the row-sum $\kappa = \sum _{n=0}^{N-1} w_{n}(0)$ and considering the vector δT to be parallel to a right eigenvector of *w*(0) we can diagonalise (12) so that the matrix in equation (14) becomes $\left[ \left( \textrm{e}^{\lambda } - 1 \right) \dot{\theta } + \kappa -\gamma _\mu (\lambda ) \right] I_N$, where $\dot{\theta } = S(\sum _{n=0}^{N-1} w_n P(A_{n,q}))$ and $I_N$ is the N×N identity matrix. For non-trivial solutions we must have that $\mathcal {E}_\mu (\lambda ) = 0$, where

37 $$\begin{aligned} \mathcal {E}_\mu (\lambda ) = \left( \textrm{e}^{\lambda } - 1 \right) \dot{\theta } + \kappa -\gamma _\mu (\lambda ) , \qquad \mu =1, \ldots , N. \end{aligned}$$

Since *w*(0) is symmetric the left and right eigenvectors are related by a transpose and we have explicitly that

38 $$\begin{aligned} \gamma _\mu (\lambda ) = \sum _{n=0}^{N-1} w_n(\lambda ) \exp \left( \frac{2 \pi \textrm{i} (\mu -1) n}{N} \right) , \end{aligned}$$

The eigenvector associated with eigenvalue $\gamma _\mu$ is given by $l^\mu = (1,a_\mu , a_\mu ^2, \ldots , a_\mu ^{N-1})/\sqrt{N}$, where $\mu =1,\ldots , N$, and $a_\mu =\exp (2 \pi \textrm{i}(\mu -1)/N)$ are the *N*th roots of unity. In this way we may determine the stability of a phase-locked state according to the condition $\text {Re}\, (\lambda ) <0$ for all μ (excluding the zero eigenvalue that arises from phase-shift symmetry).

**Fig. 3:**
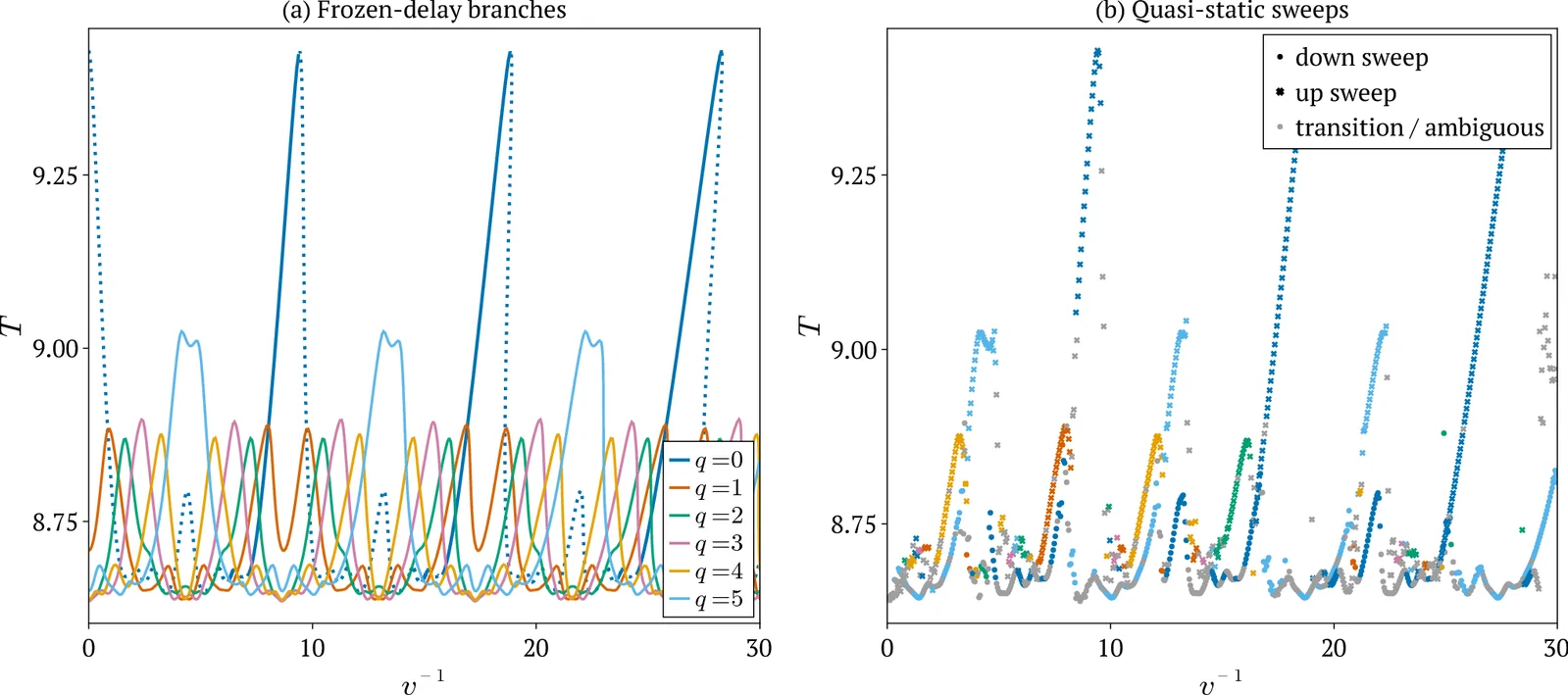
Frozen branch structure and quasi-static sweep simulations for an 11-node ring Haken network with distance-dependent delays. **a** Period *T* as a function of $v^{-1}$ for the primary twisted states q=0,1,…,5, with $w(x)=\exp (-|x|/\sigma )/(2\sigma )$. The synchronous branch q=0 is shown in dark blue: solid segments satisfy the real-eigenvalue stability condition, whereas dotted segments indicate the presence of a positive real eigenvalue. The remaining curves show the fixed-delay branches for the splay state q=1 and the twisted states q=2,3,4,5; their stability is not classified here. **b** Quasi-static sweep simulations. Circles indicate the downward sweep of $v^{-1}$ from 30 to 0, while crosses indicate the upward sweep from 0 to 30. Each point is coloured according to the nearest frozen-delay branch, using the branch colours in panel **a**; grey points indicate transitions or points for which no unambiguous nearby branch is identified. At each stage, late instantaneous ISIs are retained in small bins of $v^{-1}$. The simulations show direction-dependent tracking of the frozen branch structure, together with branch switching and hysteresis. Parameters are β=10, h=0, α=1, d=1 and σ=3

If we restrict attention to $\lambda \in \mathbb {R}$ this does not mean that $\mathcal {E}_\mu (\lambda ) \in \mathbb {R}$ since the Fourier modes occur in complex-conjugate pairs, and the modal characteristic functions are complex-valued even for real λ. However, for the synchronous state (q=0), the modal characteristic functions are real on the real axis and real-root instabilities can be detected by the sign of $\mathcal {E}_\mu (0)$. To see this note that $\lim _{\lambda \rightarrow \infty } \mathcal {E}_\mu (\lambda ) >0$ and so if $\mathcal {E}_\mu (0) < 0$ there must be a positive real root, and hence an instability. Thus for the synchronous case with $\gamma _\mu (0) \in \mathbb {R}$, a real instability occurs when $\mathcal {E}_\mu (0) = \kappa -\gamma _\mu (0)=0$ for some $\mu \ne 1$ with

39 $$\begin{aligned} \mathcal {E}_\mu (0) = \sum _{n=0}^{N-1} a_n \left[ 1 - \cos \left( \frac{2 \pi (\mu -1) n}{N} \right) \right] , \end{aligned}$$

where $a_n=a_{N-n}$ and $a_n=w_n \int _0^T S'(X_0(t)) P'(t+A_{n,0}) \textrm{d}t = w_n \sum _m Y_{-m},(\textrm{i}\omega _m) \widehat{\eta }(\omega _m)\,\textrm{e}^{-\textrm{i}\omega _m\tau _m}$ for n=0,…,N-1, where $Y_m$ are the Fourier series coefficients of $S'(X_0(t))$.

For a nonzero twisted state, the twist introduces an orientation. Even though $w_n=w_{N-n}$ and $\tau _n=\tau _{N-n}$, the coefficients contain the signed phase shift $A_{n,q}=nqT/N-\tau _n$, so generally $a_n\ne a_{N-n}$. Hence, the circulant matrix arising in the linear stability analysis is real but not symmetric. Its Fourier eigenvalues occur in complex-conjugate pairs, and $\mathcal {E}_\mu (\lambda )\in \mathbb {C}$ even when λ is real. In practice, we evaluate the λ-dependent row generator using the truncated Fourier representation described in Sect. 3. The periodised synaptic pulse and the periodic input $X_q(t)$ are reconstructed spectrally on a uniform temporal grid, and the Fourier coefficients of $S'(X_q(t))$ are obtained using a fast Fourier transform. This avoids direct numerical differentiation of the wrapped periodic pulse, which was found to be poorly conditioned. The circulant characteristic matrix is then diagonalised spatially, giving one complex scalar characteristic function $\mathcal {E}_\mu (\lambda )$ for each spatial Fourier mode μ. Candidate characteristic roots are found by solving $\operatorname {Re} \mathcal {E}_\mu (\lambda )=0$ and $\operatorname {Im} \mathcal {E}_\mu (\lambda )=0$ simultaneously from a grid of initial values in the complex λ-plane. Roots are folded into the principal strip $-\pi \le \operatorname {Im}\lambda <\pi$, duplicate solutions are removed, and the retained roots are checked by their residual and by increasing the temporal Fourier truncation. The neutral root λ=0 associated with phase-shift symmetry is excluded. Detection of any remaining root with $\operatorname {Re}\lambda >0$ establishes instability.

Figure 3 illustrates the resulting branch structure for an 11-node ring. The parameter plotted on the horizontal axis is $v^{-1}$, so moving to the right corresponds to slower conduction and hence larger distance-dependent delays. The branch curves are obtained by solving $\mathcal {H}_q(T)=0$ for the primary twisted states q=0,1,…,5. The synchronous branch is plotted with a stability diagnostic based on the real-mode condition above, using solid line segments where no real instability is detected and dashed line segments where a real instability is present. The remaining twisted branches are shown as organising branches for the fixed-delay problem.

The same figure also shows direct simulations in which $v^{-1}$ is slowly swept through the range of the bifurcation diagram. The network is first allowed to relax at the high-delay end, $v^{-1}=30$, after which $v^{-1}$ is swept down from 30 to 0 and then swept back up from 0 to 30. At each stage the instantaneous ISIs are recorded, retaining the late ISI values in small bins of $v^{-1}$ to produce a quasi-static sweep trace. Circles correspond to the downward sweep from high to low $v^{-1}$, while crosses correspond to the upward sweep from low to high $v^{-1}$. The comparison between swept simulations and frozen-delay branches shows how the network follows different attracting parts of the branch structure depending on sweep direction, with the possibility of hysteresis and branch switching. This use of a prescribed sweep is not itself an adaptive-delay model, but it anticipates the next section, where conduction speeds and delays evolve dynamically through activity-dependent white matter plasticity.

## White matter plasticity

Myelin is a fatty substance that wraps and electrically insulates axons and gives them a white colour. This *white matter* makes up roughly 50% of the human brain and is vitally important for controlling communication pathways via the modulation of axonal conduction speeds. The temporal synchrony of signals arriving from different neurons or brain regions is known to be essential for proper neural processing (Gansel 2022). Nonetheless, how this is achieved and maintained in delayed, complex networks remains incompletely understood (Pajevic et al. 2014). Experimental results show that oligodendrocytes (a type of glial cell that produces myelin) are responsive to changes in neural activity and that myelination is activity dependent. Optogenetic and electrical manipulations have shown that increases in firing activity promote myelination. Fluctuations in neural activity engage oligodendroglia, resulting in changes in axonal conduction speeds through the formation of new myelin segments, adaptive changes in myelin sheath thickness and/or internodal length (Mount and Monje 2017; Gibson et al. 2014; Xin and Chan 2020). While neuron-oligodendrocyte signalling remains poorly understood and a topic of intense research, experiments indicate that neural firing rates and axonal conduction speeds are generally positively correlated (Noori et al. 2020). Thus, there is now a consensus that white matter is *plastic*, namely that the myelination of an axon is activity dependent see e.g., (Fields 2008; Gibson et al. 2014; Vivo and Bellesi 2019). Given that myelination affects conduction speed, the delays that we have considered as fixed up until now may themselves evolve in a state-dependent fashion. The time-scale for white matter plasticity can be as short as days to weeks in the context of a new learning experience, though ranges up to years (Huber et al. 2018). This has recently begun to be explored from a modelling perspective by Lefebvre and colleagues (Noori et al. 2020; Park and Lefebvre 2020; Talidou et al. 2021, 2022; Lefebvre et al. 2025), with recent work by Fields and colleagues emphasising the role of oligodendrocyte-mediated myelin plasticity in facilitating neural synchronisation, whereby white matter plasticity may help distant brain regions remain synchronised by compensating for long transmission times through increased conduction speeds (Pajevic et al. 2014, 2023).

Building on the work of Lefebvre and colleagues it is natural to describe white matter plasticity in terms of a biologically motivated rule for the evolution of conduction speed. For a fixed axonal fibre length between nodes *i* and *j* of length $d_{ij}$, we will consider a uniformly myelinated system that gives rise to a delay $\tau _{ij}(t) = d_{ij}/c_{ij}(t)$ for a signal travelling along the fibre with speed $c_{ij}$. In the absence of any myelination we will define this speed to be $c^0_{ij}$, with a corresponding fixed delay $\tau _{ij}^0 = d_{ij}/c_{ij}^0$, such that with an increase in myelination $c_{ij} > c_{ij}^0$. To develop a plasticity rule it is important to bear in mind that i) myelination is activity dependent (via axon-glia interaction), and ii) axonal plasticity is slow and has a metabolic cost. Within the context of the spiking Haken Lighthouse model presented here we propose a phenomenological model of the form

40 $$\begin{aligned} \tau _w \frac{\textrm{d}}{\textrm{d}t} c_{ij} = c_{ij}^0 - c_{ij} + f_{ij} (a_{ij}), \end{aligned}$$

with

41 $$\begin{aligned} a_{ij} (t)&= \int _{t-\tau _{ij}(t)}^t \textrm{d}t' \, S \left( \psi _j(t') \right) ,\end{aligned}$$

42 $$\begin{aligned} f_{ij}(a)&= \kappa _{ij}\tanh (\sigma _{ij} (a-h_{ij})) . \end{aligned}$$

In this model, changes in conduction speed are governed by the interplay between two general mechanisms, namely myelin *retraction* and myelin *formation*. The activity variable $a_{ij}(t)$ integrates the firing rate of the presynaptic node *j* over the current propagation time $\tau _{ij}(t)$ of the edge from *j* to *i*. Since $\dot{\theta }_j=S(\psi _j)$, it may equivalently be interpreted as the phase advance accumulated by node *j* while a signal traverses the edge. Biologically, $a_{ij}$ is intended as a phenomenological proxy for the cumulative activity-dependent signal available to drive axon–oligodendrocyte interactions over this propagation interval; it is not intended to represent a specific molecular signalling pathway. The parameter $h_{ij}$ is an edge-dependent activity set point. When $a_{ij}>h_{ij}$, the plastic contribution $f_{ij}(a_{ij})$ is positive and promotes an increase in conduction speed, representing net myelin formation or reinforcement. When $a_{ij}<h_{ij}$, the plastic contribution is negative and promotes a reduction in conduction speed, representing net myelin retraction or weakening. Thus $f_{ij}$ is allowed to be negative. The parameter $\sigma _{ij}$ controls the sensitivity of the plastic response to deviations from the set point: small $\sigma _{ij}$ gives a gradual response, whereas large $\sigma _{ij}$ gives a sharp, switch-like transition. The parameter $\kappa _{ij}$ sets the maximum magnitude of the activity-dependent contribution, since $-\kappa _{ij}\le f_{ij}(a)\le \kappa _{ij}$. The phenomenological rule does not intrinsically impose a positivity constraint on $c_{ij}$. If one wishes to guarantee positive conduction speeds for all time, a sufficient condition is $c_{ij}(0)>0$ together with $0\le \kappa _{ij}<c_{ij}^0$. Indeed, at the boundary $c_{ij}=0$ the plasticity rule then satisfies $\tau _w\dot{c}_{ij}=c_{ij}^0+f_{ij}(a_{ij})\ge c_{ij}^0-\kappa _{ij}>0$, so the positive-speed region is forward invariant. Finally, $\tau _w$ sets the slow timescale of white-matter plasticity relative to the fast neuronal dynamics.

A useful form for $a_{ij}$ that can be readily implemented numerically is obtained by differentiation as

43 $$\begin{aligned} \frac{\textrm{d}}{\textrm{d}t} a_{ij} (t) = S \left( \psi _j(t) \right) - S \left( \psi _j(t-\tau _{ij}(t)) \right) \left[ 1- \frac{\textrm{d}}{\textrm{d}t} \tau _{ij} (t) \right] \end{aligned}$$

with initial data $a_{ij} (0) = \int _{-\tau _{ij}(0)}^0 \textrm{d}t \, S \left( \psi _j(t) \right)$ (if the history is specified for t≤0). In contrast to previous white matter plasticity rules for phase-oscillator networks as in Karimian et al. (2019); Park and Lefebvre (2020) the evolution for speed is governed by a state-dependent delay differential equation as opposed to an ordinary differential equation (ODE).

The next subsection exploits the time-scale separation between the fast spiking dynamics and the slow conduction-speed dynamics to interpret this adaptive system from a slow–fast perspective.

### A *slow–fast* perspective

The adaptive system combines fast spiking dynamics with slow changes in conduction speed. It is therefore natural to view it as a slow–fast problem, with the static-delay theory of the previous sections providing the frozen fast subsystem. Writing *X* for the collection of fast Lighthouse variables $(\theta _i, \psi _i, \dot{\psi }_i)$ and *c* for the matrix of conduction speeds ($[c]_{ij}=c_{ij}$), the full adaptive system takes the abstract form

44 $$\begin{aligned} \dot{X} = F(X;c), \qquad \tau _w \dot{c} = G(X,c), \end{aligned}$$

where the delays entering the fast subsystem are slaved to the conduction variables through $\tau _{ij}=d_{ij}/c_{ij}$. Introducing $\varepsilon =\tau _w^{-1}\ll 1$ gives the singularly perturbed form

45 $$\begin{aligned} \dot{X} = F(X;c), \qquad \dot{c} = \varepsilon G(X,c). \end{aligned}$$

Thus the spiking dynamics evolves on the fast timescale while the conduction variables drift through delay space on the slow timescale. It is worth stressing that the fast subsystem is not a typical smooth ODE flow. Rather, it is naturally formulated in terms of event times and spike trains (and the linear stability theory developed earlier is correspondingly written in terms of perturbations of firing times). This event-driven structure is one of the attractive features of the Lighthouse model, but it also means that some of the standard hypotheses and proofs from geometric singular perturbation theory for smooth finite-dimensional flows cannot be imported wholesale (Jones 1995). In particular, when speaking of critical manifolds, normal hyperbolicity, and slow manifolds below, one should understand these statements at a formal or geometric level, guided by the spectral stability theory of the frozen event-driven problem. A fully rigorous invariant-manifold theory for the adaptive event-driven system would require additional work, especially when recognising that the slow flow is governed by a state-dependent delay differential equation.

For ε=0 the conduction variables are frozen, and the fast subsystem reduces to the static-delay Lighthouse problem analysed earlier (and parameterised by *c*). A branch of frozen phase-locked states therefore defines a critical manifold

46 $$\begin{aligned} \mathcal {M}_0=\{(X^*(\cdot ;c),c): c \text { lies on the branch}\}, \end{aligned}$$

in the extended fast–slow state space. Here $X^*(\cdot ;c)$ denotes the corresponding frozen phase-locked orbit. In this case the activity variable $a_{ij}$ depends on how the delay $\tau _{ij}$ compares with the period *T*. If

47 $$\begin{aligned} \tau _{ij}=(p_{ij}+r_{ij})T, \qquad p_{ij}\in \mathbb {Z}_{\ge 0}, \qquad r_{ij}\in [0,1), \end{aligned}$$

then the integration window in (41) spans $p_{ij}$ full periods together with a fractional interval of length $r_{ij}T$, so that

48 $$\begin{aligned} a_{ij}(t)=2\pi p_{ij}+\int _{t-r_{ij}T}^{t} \textrm{d}t'\, S\big (\psi _j(t')\big ). \end{aligned}$$

An especially interesting case is the integer family of commensurate states (e.g., the asymmetric branches of Sect. 5):

49 $$\begin{aligned} \tau _{ij}=p_{ij}T, \qquad p_{ij}\in \mathbb {Z}_{\ge 0}, \end{aligned}$$

for which the residual term in (48) vanishes and hence

50 $$\begin{aligned} a_{ij}(t)=2\pi p_{ij}. \end{aligned}$$

Thus, the slow adaptive law closes exactly on the corresponding phase-locked branch to yield the autonomous ODE:

51 $$\begin{aligned} \tau _w \dot{c}_{ij} = c_{ij}^0-c_{ij}+\kappa _{ij}\tanh \!\big (\sigma _{ij}(2\pi p_{ij}-h_{ij})\big ). \end{aligned}$$

Thus the nonlinear plasticity rule retains the special role of commensurate phase-locked states, but the adaptive forcing now depends on the signed mismatch $2\pi p_{ij}-h_{ij}$.

The relevant notion of normal hyperbolicity is inherited from the event-driven linear stability problem for the frozen phase-locked state. After quotienting out the neutral direction associated with global phase shift, a branch is normally attracting if every non-trivial fast exponent satisfies $\text {Re}\,\lambda <0$, with λ determined as $\mathcal {E}(\lambda )=0$ as described in Sect. 3. When this holds, it is natural to regard $\mathcal {M}_0$ as a normally attracting critical manifold. Formally, and in the spirit of geometric singular perturbation theory, one then expects that for sufficiently small ε there exists a nearby locally invariant slow manifold $\mathcal {M}_\varepsilon$ that perturbs smoothly from $\mathcal {M}_0$. Trajectories first relax rapidly toward $\mathcal {M}_\varepsilon$ in the fast directions and then drift slowly along it under the induced adaptive dynamics.

A commensurability condition such as (49) is not by itself sufficient to define an adaptive steady state. One must also satisfy the kinematic relation $\tau _{ij} = d_{ij}/c_{ij}$ and the frozen phase-locking conditions that determine the relevant branch T=T(c). A fixed point $c^*$ of the reduced slow flow on an integer-commensurate branch therefore satisfies

52 $$\begin{aligned} c_{ij}^*=c_{ij}^0+\kappa _{ij}\tanh \!\big (\sigma _{ij}(2\pi p_{ij}-h_{ij})\big ), \end{aligned}$$

together with the compatibility condition

53 $$\begin{aligned} \frac{d_{ij}}{c_{ij}^*}=p_{ij}T(c^*). \end{aligned}$$

Hence the existence of a slow fixed point on a $p_{ij}T$ family is a joint condition involving the frozen phase-locked branch, the anatomical distances $d_{ij}$, and the plasticity parameters $(c_{ij}^0,\kappa _{ij},\sigma _{ij},h_{ij})$. This highlights that a candidate adaptive phase-locked state is selected not merely by the existence of a static phase-locked solution, but by a mutual compatibility between the fast branch geometry and the nonlinear plasticity rule. In particular, the thresholds $h_{ij}$ can be used to bias the slow dynamics toward some commensurability classes and away from others.

When (49) does not hold exactly, the quantity $a_{ij}(t)$ retains an explicit dependence on the phase of the fast orbit through (48). On a normally attracting branch of frozen phase-locked states one may then pass to a reduced slow flow on the corresponding slow manifold. This gives rise to a non-autonomous ODE that can be simplified to an autonomous form after realising that *averaging* can be legitimately applied. Averaging is suitable when there is a clear timescale separation, so that the fast spiking dynamics settles rapidly onto a frozen *T*-periodic phase-locked orbit while the conduction variables change only by O(ε) over one fast cycle. In that setting, the slow variables effectively see only the cycle-averaged forcing from the fast subsystem with $a_{ij}(t) = a_{ij} (t+ T(c))$. If $X^*(t;c)$ denotes the corresponding frozen *T*(*c*)-periodic orbit, then the averaged reduced slow flow is

54 $$\begin{aligned} \tau _w \dot{c}_{ij} = c_{ij}^0-c_{ij} + \frac{\kappa _{ij}}{T(c)}\int _0^{T(c)} \tanh \!\big (\sigma _{ij}(a_{ij}(t;c)-h_{ij})\big ) \textrm{d}t. \end{aligned}$$

A slow fixed point $c^*$ on the branch therefore satisfies

55 $$\begin{aligned} c_{ij}^*=c_{ij}^0+ \frac{\kappa _{ij}}{T(c)}\int _0^{T(c)} \tanh \!\big (\sigma _{ij}(a_{ij}(t;c^*)-h_{ij})\big ) \textrm{d}t. \end{aligned}$$

together with the requirement that $c^*$ lie on the frozen phase-locked branch under consideration.

Linearising the exact reduced slow flow (54) at a reduced slow fixed point gives

56 $$\begin{aligned} \tau _w {\dot{\eta }} = \textrm{D}F(c^*)\eta , \end{aligned}$$

where

57 $$\begin{aligned} [\textrm{D}F(c)]_{(ij),(k\ell )}&=-\delta _{ik}\delta _{j\ell } \nonumber \\&\quad +\frac{1}{T(c)}\int _0^{T(c)} f'_{ij}\!\big (a_{ij}(t;c)\big ) \frac{\partial a_{ij}}{\partial c_{k\ell }}(t;c)\textrm{d}t, \end{aligned}$$

and $f'_{ij}(a)=\kappa _{ij}\sigma _{ij}\operatorname {sech}^2\!\big (\sigma _{ij}(a-h_{ij})\big )$. The factor $f'_{ij}(a)$ controls how strongly each edge responds to perturbations. Edges operating near threshold are highly plastic, whereas edges in a saturated regime are effectively pinned. The full adaptive state is attracting when the frozen phase-locked orbit is spectrally stable in the fast subsystem and the reduced slow fixed point is stable within the slow manifold. We note that for the commensurate states given by (49) the slow flow is given exactly by (51) and since $\partial a_{ij} / \partial c_{k\ell }(t;c) = 0$ along the exact commensurate manifold where $a_{ij}=2\pi p_{ij}$ (so that $\textrm{D}F$ only has eigenvalues -1) steady states in the slow subsystem are stable (though may be unstable in the fast subsystem).

If a reduced slow fixed point is stable along the slow manifold but the frozen phase-locked orbit is unstable in a fast direction, then it can act as an organising saddle for the full dynamics. Trajectories may be drawn toward it in conduction space and linger near it for long times, but are ultimately expelled in the transverse fast directions. Such states naturally organise switching, bottlenecks, and delayed escape. The reduced slow description is valid only while the underlying branch remains normally hyperbolic. When the frozen fast subsystem loses stability (for example at a fold, a symmetry-breaking point, or a dynamic instability) normal hyperbolicity fails and the reduced single-manifold description breaks down. One then expects a combination of slow drift along the attracting portion of a branch and fast jumps to other branches. In this way the geometric singular perturbation viewpoint naturally accommodates convergence to a stable adaptive state, switching between competing phase-locked states, and delayed departure from weakly unstable states. The nonlinear plasticity rule also suggests a natural mechanism for relaxation oscillations. Suppose there are two competing phase-locked branches, each normally attracting for the frozen fast subsystem over some interval, but such that the induced slow flow along one branch points toward larger delays while the induced slow flow along the other points toward smaller delays. Then the system may drift slowly along the first branch until normal hyperbolicity is lost, undergo a rapid jump to the second branch, drift slowly back in the opposite direction, and repeat. This produces a slow–fast alternation between branchwise drift and fast transitions, i.e. a relaxation oscillation in conduction (or delay) space. By contrast, if the slow flow does *not* reverse direction between nearby stable branches, then persistent switching is less likely. In that situation trajectories may instead approach a final slow steady state, either on an exactly commensurate branch satisfying (52)–(53) or on a nearby non-commensurate branch satisfying (55). Thus the nonlinear rule supports both possibilities: sustained slow switching when branchwise drifts oppose one another, and convergence to a final adaptive state when the slow drift is globally directed toward a compatible attractor.

At the geometric level, the relaxation mechanism discussed above for slow oscillations is the same as that described by Klinshov and Nekorkin for a neuronal oscillator with adaptive delayed feedback (Klinshov and Nekorkin 2024). In both models, the adaptive delay evolves on a slow timescale, the trajectory drifts along attracting branches of the corresponding frozen-delay system, and the loss of an attracting branch produces a rapid transition to another branch. Repetition of this slow drift and fast transition generates oscillations on the plasticity timescale that are absent when the delay is fixed. Although the mechanisms are the same in their broad slow–fast organisation, there are differences in the underlying models and adaptation rules. Klinshov and Nekorkin consider a single oscillator with adaptive delayed feedback, whereas the present study concerns general networks of Lighthouse neurons with directed conduction speeds. Here the slow forcing is determined by presynaptic activity integrated over the current propagation interval, and the fast transitions occur between collective phase-locked states, including synchronous, anti-synchronous and asymmetric states.

### Numerical illustrations

Here we revisit the study from Sect. 5 with fixed delays and allow them to become plastic according the white matter plasticity rule introduced above. With the introduction of the plasticity rule (40)–(42) we use this to illustrate two key observations concerning i) the evolution to a stationary phase-locked state, and ii) the opportunity for oscillations that switch between states. To make a comparison between theory and simulations it is useful to complement the notion of a firing event with a firing phase. We can generate a phase associated with the firing times of each neuron as follows. Let $$\mathcal {T}_i^m$$ be the set of firing times lying in the interval $$[T_i^m, T_i^{m+1})$$. If there is only one spike in this interval, we transform it into a phase $$\phi _i^m \in [0,1)$$ using the transformation (Pinsky and Rinzel 1995)

58 $$\begin{aligned} \phi _i^m=\frac{\mathcal {T}_i^m-T_i^m}{T_i^{m+1} - T_i^m}. \end{aligned}$$

If the interval contains no spike or more than one spike, a unique relative phase cannot be assigned by this definition and no phase value is recorded for that interval.

In the earlier reciprocal two-node problem presented in Sect. 5, the frozen branch diagrams are computed on the equal-delay subspace $\tau _{12} = \tau _{21}= \tau$. We note that (for the reciprocal pair) the averaged slow flow given by (54) is symmetric under exchange of the two directed edges provided the edge parameters are matched and the frozen fast branch is itself symmetric (namely the synchronous and anti-synchronous solutions). A similar statement holds for slow flow on the asymmetric commensurate branch as long as we impose the further condition that $p_{12}=p_{21}$. For the purposes of using Fig. 2 as a backbone for organising the full dynamics with white matter plasticity we shall restrict ourselves to this subspace.

**Fig. 4:**
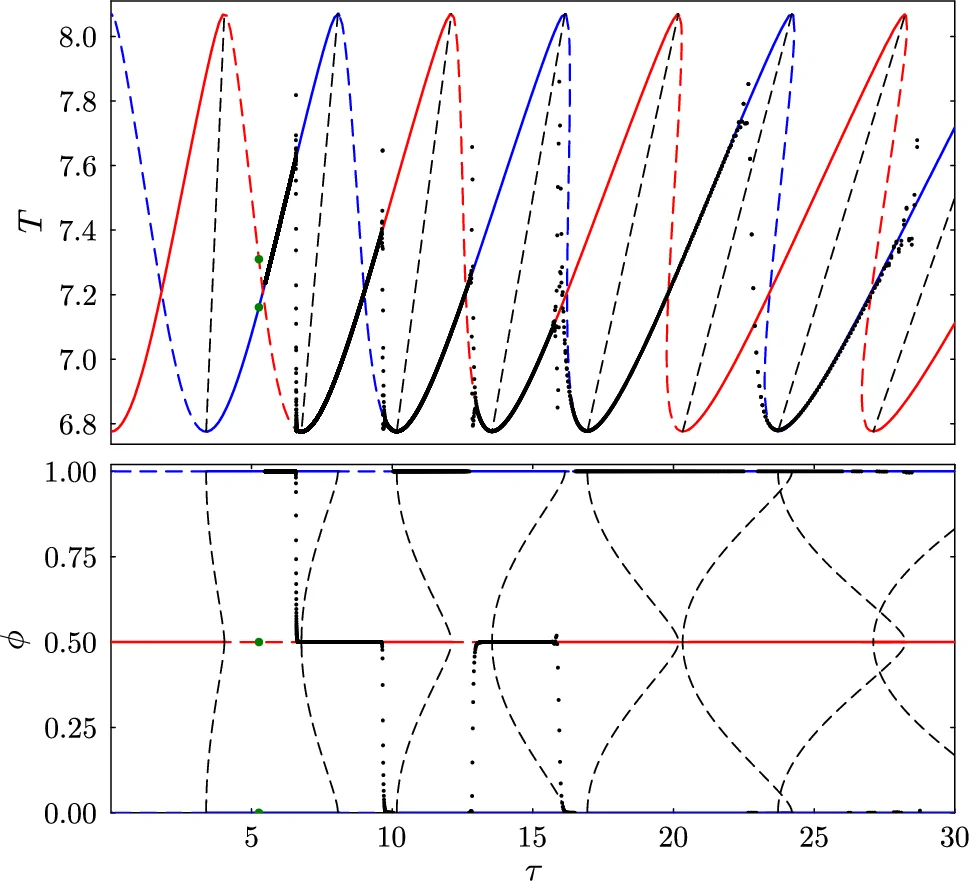
Numerical simulation of the reciprocal two-node Haken model with white matter plasticity on the symmetric subspace $\tau _1=\tau =\tau _2$, where $\tau _1=\tau _{12}$ and $\tau _2=\tau _{21}$. The upper panel shows instantaneous ISIs for both neurons against the current delay τ at firing events, and the lower panel shows the corresponding instantaneous phase defined by (58). The coloured curves are the frozen-delay branches from the two-node bifurcation diagram Fig. 2, while black points show the adaptive simulation projected onto the same coordinates. Filled green circles mark fixed points of the reduced slow flow on symmetric branches. The simulation shows rapid attraction toward a phase-locked state, slow drift along the frozen branch structure, switching between stable symmetric branches, and final approach to a slow fixed point. Parameters are as in Fig. 2, with $d_{12}=1=d_{21}$, $\tau _{12}^0=5=\tau _{21}^0$, $\kappa _{12}=0.01=\kappa _{21}$, $h_{12}=8\pi =h_{21}$, $\sigma _{12}=1=\sigma _{21}$, and $\tau _w=10^4$. The initial delay history is constant with $\tau _{12}(0)=30=\tau _{21}(0)$

In Fig. 4 we show a simulation of the full adaptive system on the symmetric subspace $\tau _1 = \tau = \tau _2$. Here we plot the instantaneous ISI (for both neurons) and the corresponding value of τ at a firing event in the upper panel and a similar plot using instantaneous phase in the lower panel. The coloured curves show the frozen-delay branches from the two-node bifurcation diagram, while the black simulation points show the trajectory of the adaptive system projected onto this frozen branch structure. The trajectory first relaxes rapidly toward a phase-locked state and then evolves slowly as the conduction delay changes. During this slow drift it switches between stable symmetric branches before approaching a fixed point of the reduced slow flow, indicated by a filled green marker. This behaviour is consistent with the slow–fast picture: the adaptive dynamics is organised by attracting pieces of frozen phase-locked branches, while the slow plasticity rule moves the system through delay space.

**Fig. 5:**
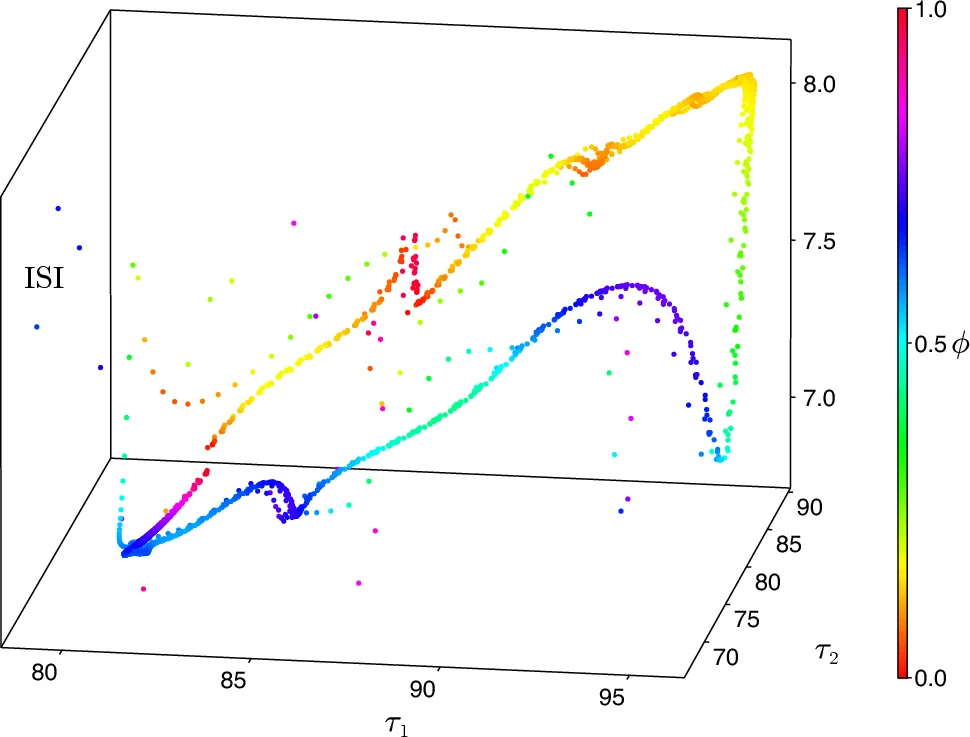
Numerical simulation of the reciprocal two-node Haken model with two plastic delays showing $(\text {ISI},\tau _1,\tau _2)$. The colour of data points indicates the corresponding instantaneous phase. The simulations show switching between low and high ISIs. Parameters are the same as in Fig. 4 with $(\kappa _{12},\kappa _{21}) = (0.1,0.1)$, $(\tau _{12}^0, \tau _{21}^0) = (90,80)$, and $(h_{12},h_{21})=(74.4,69.8)$

Given that multi-stability of solutions increases with higher values of a frozen τ (on the subspace $\tau _1 = \tau = \tau _2$) it is of interest to explore switching behaviour for $\tau _{12}^0 \ne \tau _{21}^0$ with both values high. An example of behaviour that can emerge in this parameter regime is shown in Fig. 5. Here, we report the triple $(\text {ISI},\tau _1,\tau _2)$ and colour code points according to the value of the instantaneous phase. We see a loose loop of data points that switches between low and high ISIs in a repetitive fashion. This is the numerical signature of the relaxation-type scenario described in Sect. 7.1: slow drift along one attracting branch is followed by a faster transition to another branch, after which the slow drift proceeds in a different direction.

The mechanism for generating such switching behaviour seems to be most robust when the thresholds $h_{ij}$ are centred around values in the vicinity of a commensurate state with $a_{ij} = 2 \pi p_{ij}$ with $p_{ij}\in \mathbb {Z}_{\ge 0}$. With such a choice there is a good opportunity for $a_{ij}(t) - h_{ij}$ to change sign and reverse the direction of the slow flow after a jump between fast solution branches, providing a natural route to switching and long-time alternation between distinct phase-locked states rather than settling directly to a single compatible fixed point. The simulation in Fig. 5 should therefore be interpreted not as a generic outcome for all parameters, but as an illustration of how the nonlinear plasticity rule can organise recurrent switching when the thresholds place the system near competing commensurability classes.

Together, these examples show how the static-delay bifurcation diagrams continue to organise the adaptive system. When the slow drift is directed toward a compatible fixed point, the dynamics converges to a stationary phase-locked state. When nearby branches induce opposing slow drifts and the fast subsystem allows transitions between them, the same plasticity rule can generate long-time alternation between distinct phase-locked states. This motivates the next subsection, where the thresholds in the plasticity rule are used more deliberately to bias a larger network toward commensurate conduction delays.

### Sculpting a commensurate state

For an arbitrary network with heterogeneous choices of fixed weights $w_{ij}$ and delays $\tau _{ij}$, one would not expect the spontaneous emergence of a frequency locked state in which all nodes share a common firing frequency. Nevertheless, the white matter plasticity rule introduced above provides a natural mechanism for biasing the system toward a commensurate delay structure. The key observation is that the activity variable $a_{ij}$ associated with the directed edge from node *j* to node *i* samples the firing activity of the presynaptic node *j* over a time window of length $\tau _{ij}$. Thus the relevant clock for choosing a commensurability class on this edge should be the intrinsic or emergent period of the source node *j*, rather than a single externally imposed network period.

This suggests a more general sculpting principle. Rather than trying to impose a global rhythm directly, the adaptive rule can be used locally to make each axonal pathway sensitive to whether its conduction delay spans an integer number of cycles of the activity arriving from its source. In biological terms, the threshold on a given fibre should reflect the typical amount of presynaptic activity expected over one, two, or more cycles of the source neuron. In algorithmic terms, the network is first allowed to reveal the characteristic timescale of each node, and the plasticity thresholds are then chosen so that each edge is most responsive near a delay that is commensurate with the timescale of the node that drives it. The aim is not to prescribe a final phase relationship, but to bias the admissible delay landscape so that mutually compatible phase-locked states become easier for the fast dynamics to select.

**Fig. 6:**
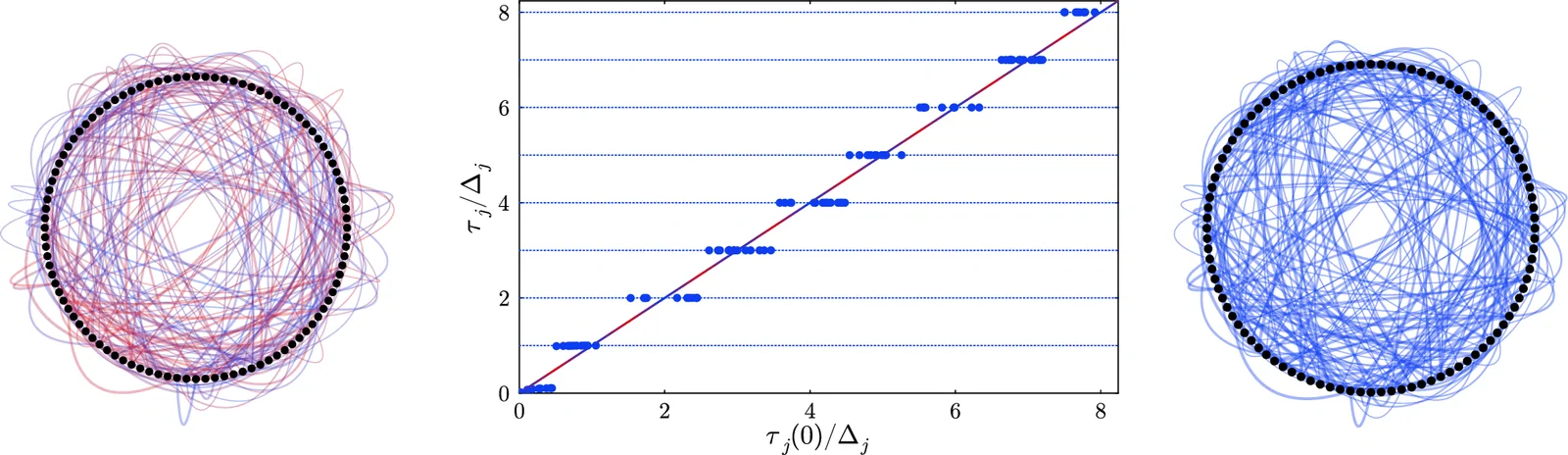
Schematic and quantification of node-wise commensurability classes for a randomly connected Haken network with N=100. Left: before adaptation, fibres are coloured by their delay-period mismatch, measured as the distance of $\tau _j(0)/\Delta _j$ from the nearest integer. Blue indicates commensurate delays and red indicates half-integer mismatch. Middle: for each source node *j*, a target period $\Delta _j$ is estimated from the median inter-spike interval, and the integer class $p_j=\operatorname {round}(\tau _j(0)/\Delta _j)$ is assigned. Blue points show the resulting discrete classes, blue horizontal guide lines mark integer values of $p_j$, and the diagonal is coloured by the periodic mismatch map. Right: after delay sculpting, fibres are coloured by their residual mismatch after assignment to the commensurate class, so that edges lie on blue delay-period classes. Weights are drawn independently from a uniform distribution on [-1/N,1/N], with self-connections excluded, and initial delays are drawn from a uniform distribution on (0, 100]. Parameters are α=1, β=10, h=0, d=1, $\sigma _j=1$ and $\kappa _j=1$ for all *j*

One concrete realisation of this principle is to estimate a node-wise target period from the typical inter-spike interval of each source node. For example, we may run the frozen-delay network, or use an early weakly adapted epoch, and record the firing times of each node. We define

59 $$\begin{aligned} \Delta _j = \operatorname {median}_m \Delta _j^m, \qquad \Delta _j^m = T_j^{m+1} - T_j^m . \end{aligned}$$

For each directed edge (*i*, *j*) we then assign an integer commensurability class by comparing the initial delay on that edge with the target period of the presynaptic node,

60 $$\begin{aligned} p_{ij} = \operatorname {round} \left( \frac{\tau _{ij}(0)}{\Delta _j} \right) = \operatorname {round} \left( \frac{d_{ij}}{c_{ij}(0)\Delta _j} \right) . \end{aligned}$$

We also choose the baseline conduction speed in the plasticity rule to coincide with the initial conduction speed, $c_{ij}^0 = c_{ij}(0)$. With this convention the unadapted network provides the reference state about which myelination acts. In the absence of activity-dependent modulation the conduction speeds relax back to their initial values, so any subsequent drift in delay space is generated by the plastic component of the rule rather than by an imposed shift of the baseline architecture. The corresponding threshold is chosen as $h_{ij}=2\pi p_{ij}$.

This choice is motivated by the exact commensurate identity discussed above: if the presynaptic node *j* is phase-locked with period $\Delta _j$ and the delay satisfies $\tau _{ij}=p_{ij} \Delta _j$, then the activity integral over the propagation window accumulates precisely $p_{ij}$ cycles of firing activity, giving $a_{ij}=2\pi p_{ij}$. Hence $h_{ij}=2\pi p_{ij}$ places the edge close to the switching surface of the tanh nonlinearity when the delay is near the desired commensurability class.

In this construction the plasticity rule does not prescribe the phase differences between nodes. Rather, it makes each outgoing edge from node *j* most sensitive when its delay is close to an integer multiple of the typical period of node *j*. The gains $\kappa _{ij}$ and sharpnesses $\sigma _{ij}$ then control how strongly and how abruptly the edge responds to departures from this commensurate configuration. Large $\sigma _{ij}$ makes the plasticity act almost like an edgewise switch, whereas more moderate values provide a smoother and more robust drift in delay space. In this way the adaptive rule sculpts the network toward a set of node-wise commensurate delays, leaving the final phase-locked pattern to be selected by the fast Haken dynamics.

The estimated period $\Delta _j$ is used only to assign the initial integer class $p_{ij}$ and hence the threshold $h_{ij}=2\pi p_{ij}$; it is not subsequently imposed as a fixed target period. During adaptation, $a_{ij}(t)$ is evaluated from the actual evolving activity of the presynaptic node over the current propagation interval. Thus moderate changes in firing period are incorporated into the plasticity signal itself. If node *j* remains approximately periodic with a slowly varying period, $a_{ij}$ continues to measure the activity accumulated over approximately $\tau _{ij}/\Delta _j(t)$ cycles, and the class $p_{ij}$ retains its interpretation as the desired number of cycles spanned by the delay. Nevertheless, $p_{ij}$ is fixed in the present construction. If the period changes sufficiently that a neighbouring integer class becomes more appropriate, the original assignment need no longer be optimal. The activity integral remains well-defined when the presynaptic spike train is irregular or jittering, so the plasticity dynamics can still be simulated without assigning an instantaneous period at every time. The initial class assignment, however, requires a representative timescale. A median ISI is robust to occasional anomalous intervals when the ISI distribution is concentrated around a typical value. For strongly non-stationary activity, or for a broad or multimodal ISI distribution, a single median period and hence a unique one-shot class assignment may not be meaningful. Such cases lie outside the scope of the present construction. A possible extension would use a running robust estimate of the local period and allow hysteretic reassignment between neighbouring integer classes.

To reduce the number of state variables we shall consider networks with a fixed distance *d* between all nodes and a common speed on all edges emanating from node *i*. In this way we need only consider *N* distinct speeds with associated delays $\tau _i= d/c_i$. The white matter plasticity rule (40)-(42) then reduces to the *N* (as opposed to $N^2$) equations

61 $$\begin{aligned} \begin{aligned} \tau _w \frac{\textrm{d}}{\textrm{d}t} c_i&= c_i^0-c_i + \kappa _i \tanh (\sigma _i (a_i - h_i)), \\ a_i(t)&= \int _{t-\tau _i(t)}^t \textrm{d}t' S(\psi _i(t')) , \qquad i=1,\ldots ,N . \end{aligned} \end{aligned}$$

Fig. 6 shows the result of applying this construction to a randomly connected Haken network. Here, the weights $w_{ij}$ are quenched random variables drawn from a uniform distribution on [-1/N,1/N]. The horizontal axis shows the initial delay of each source node, normalised by its measured target period, while the vertical axis shows the corresponding integer $p_j$ selected by rounding. The blue points lie on horizontal integer levels, and the coloured diagonal indicates the delay-period mismatch, measured as the distance of $\tau _j(0)/\Delta _j$ from the nearest integer. Blue indicates commensurate delays and red indicates half-integer mismatch. The figure confirms that the rule partitions the heterogeneous set of initial delays into discrete commensurability classes. These classes do not impose a specific phase-locked state. Instead, they define the delay values at which the plasticity rule is most sensitive, thereby shaping the slow drift of the network toward conduction delays compatible with the characteristic activity timescales of the source nodes.

From a neuroscientific perspective, a plasticity rule that drives conduction delays into commensurability classes suggests that finite transmission delays need not destroy the phase of an oscillatory signal. If the delay on an outgoing line from node *j* satisfies $\tau _j \approx p\,T_j$, for some integer *p* and characteristic period $T_j$ of the oscillation generated by that node, then the delayed signal returns to approximately the same phase point on the sender’s cycle. In this sense, the delay becomes *phase-preserving*: the receiver is driven by an input whose phase still reflects the state of the sending oscillator modulo 2π. Adaptive myelination may therefore be understood not simply as a mechanism for increasing conduction speed, but as a way of tuning transmission delays so that rhythmic phase information is preserved along long-range pathways (Fries 2015; Fields 2015; Pajevic et al. 2014). This interpretation is closely aligned with the communication-through-coherence hypothesis, according to which the efficacy of interaction between neuronal populations depends on the phase at which inputs arrive (Fries 2015; Schoffelen et al. 2005). A delay that is commensurate with the sender’s oscillation period preserves that phase relation up to an integer number of cycles, thereby organising pathways into discrete timing classes. The significance of such a mechanism is not necessarily that it produces a dramatic increase in global synchrony, but that it allows long-range coupling to remain temporally meaningful despite substantial delays. This is consistent with the broader literature on activity-dependent myelination, which suggests that adaptive changes in myelin can regulate conduction velocity, synchrony, and the temporal coordination of distributed circuits (Fields 2015; Pajevic et al. 2014; Mount and Monje 2017), and with experimental evidence that regional myelination can be adjusted so as to preserve timing relationships across pathways of different lengths (Salami et al. 2003). In this view, the main function of a plasticity rule that drives delays into commensurability classes is to render transmission lines approximately phase-transparent, so that oscillatory information can be conveyed without being degraded by the delay itself.

## Discussion

The aim of this paper has been to develop a tractable framework for studying phase-locked states in spiking Haken Lighthouse networks with conduction delays, and then to use this fixed-delay theory as the organising backbone for a model of activity-dependent white matter plasticity. The starting point is the observation that the Lighthouse model occupies a useful middle ground between detailed spiking descriptions and rate-based neural models: spikes remain the elementary events of communication, but the event-time structure is sufficiently explicit to permit analytical progress. Building on Haken’s original formulation of pulse-coupled neural activity (Haken 2002) and the recent revisiting of the Lighthouse model (Coombes 2025), we have shown how multiple delays can be incorporated into the existence and stability theory for phase-locked states.

The first contribution is the fixed-delay phase-locking theory. For general networks we derived implicit equations for phase-locked states and an associated spectral condition for their linear stability, formulated directly in terms of perturbations of firing times. This event-time viewpoint is well matched to a spiking model: stability is determined by how small perturbations to one generation of spikes affect subsequent generations. The approach was illustrated first for a single delayed autapse, where regular-spiking branches, folds and dynamic instabilities can be identified. In this minimal setting the loss of stability naturally produces modulated inter-spike intervals, providing a mathematically transparent route to jittering of spike trains. For reciprocal two-node networks, the same formalism separates synchronous, anti-synchronous and asymmetric phase-locked states, and shows how asymmetric branches attach to extrema of symmetric branches. For ring networks with distance-dependent delays, the circulant structure permits a modal decomposition and gives a direct route to the stability of synchronous, splay and twisted states.

The examples developed here show that conduction delays can act as bifurcation parameters, stability selectors and adaptive state variables, rather than simply as fixed transmission lags. In neural systems, conduction delays arise from finite axonal propagation speeds and synaptic filtering, and can be comparable to the timescales of rhythmic activity. In artificial or bio-inspired systems, delayed communication is likewise unavoidable whenever signals propagate through a spatially extended substrate. The present analysis shows that such delays can shape not only the period of a network rhythm, but also the stability, multiplicity and switching of coherent spiking states. Thus delays should be regarded as an active component of the network dynamics, rather than ignored or absorbed into an instantaneous coupling approximation.

The second contribution is to turn this viewpoint into an adaptive theory. Motivated by activity-dependent myelination, we introduced a phenomenological plasticity rule in which conduction speeds evolve slowly in response to activity over the propagation window of a fibre. Since the delays are slaved to the conduction speeds, this produces a spiking network with state-dependent delays. The fixed-delay bifurcation structure then becomes a frozen fast subsystem for a slow–fast adaptive problem. In this interpretation, the branch diagrams obtained for static delays form an organising skeleton for the full adaptive dynamics: trajectories can drift along stable phase-locked branches, approach slow fixed points, switch between competing branches, or undergo relaxation-type alternations when the slow flow reverses direction between them.

A particularly simple mechanism emerges near commensurate delay–period relationships. When the propagation time along an edge contains an integer number of cycles of the sending node, the activity accumulated over that propagation window closes over complete periods and no longer depends on the phase at which the window is opened. The plasticity rule therefore has a natural local reference point: thresholds can be chosen so that each edge is most sensitive near a prescribed integer delay–period class. In this way, the same local rule can bias different edges toward different commensurate timing relationships. In the reduced node-wise example of Sect. 7.3, this mechanism partitions outgoing connections into discrete commensurability classes. Biologically, such a mechanism suggests that adaptive myelination may help preserve the phase of rhythmic signals over long-range connections. This is naturally related to communication-through-coherence ideas (Fries 2015) and to the broader literature on myelin plasticity and neural synchronisation (Pajevic et al. 2014, 2023). It also complements recent work on the effect of white matter delays on large-scale brain synchrony, where the delay structure of white matter pathways is treated as a key determinant of collective rhythmic behaviour (Şayli and Coombes 2024).

From the perspective of network computation, the results suggest a route by which white matter could reshape the attractor landscape of a spiking network. Phase-locked states provide candidate network states, while delay-induced changes in stability and accessibility provide possible transitions between them. The adaptive rule studied here does not prescribe a global target state, nor does it guarantee convergence to synchrony. Instead, it acts locally on edges or outgoing fibres and thereby changes the timing relations that determine which phase-locked states are stable, metastable or reachable. In this sense, the model supports a cybernetic interpretation of white matter: conduction delays are regulated variables that can shape the timing, stability and transitions of distributed neural activity. This is consistent with the broader idea that network attractors may be sculpted by modifying the communication delays between oscillatory nodes, rather than only by changing synaptic weights (Kori and Kuramoto 2001; Ashwin et al. 2024).

There are several limitations to the present study. The white matter plasticity rule is deliberately phenomenological. It captures the idea that myelination is activity dependent and slow, but it does not attempt to resolve the cellular mechanisms of axon-glia signalling, sheath formation, internodal length adaptation or metabolic cost (Mount and Monje 2017; Xin and Chan 2020; Nishiyama et al. 2021; Saab et al. 2013; Tepavčević 2021). Similarly, each connection is represented by a single effective conduction speed, whereas real white matter pathways contain heterogeneous axons and distributed conduction properties. The slow–fast interpretation should also be understood as a geometric organising principle rather than a rigorous Fenichel-type invariant-manifold theorem (Fenichel 1971). The fast subsystem is event driven, and the adaptive model contains state-dependent delays, so the usual hypotheses of smooth finite-dimensional geometric singular perturbation theory do not apply directly. Finally, most of the examples studied here are low-dimensional or highly structured. This is intentional: they are chosen to expose mechanisms. Extending the framework to heterogeneous connectome-like networks will require further analytical and numerical development.

A first direction for future work is therefore to place the slow–fast picture on firmer mathematical foundations. It would be valuable to develop an invariant-manifold and averaging theory for event-driven spiking systems with slowly evolving state-dependent delays, using the firing-time spectral problem as the replacement for the usual Floquet theory of smooth flows. A second direction is computational. Networks with many plastic delays will require continuation tools capable of tracking phase-locked branches, locating folds and dynamic instabilities, and following changes in stability as multiple conduction speeds evolve. The event-time formulation used here suggests one promising route for such algorithms, especially when combined with modern numerical continuation methods for delay equations (Engelborghs et al. 2000; Sieber et al. 2014). A complementary approach is provided by harmonic balance methods for rate-based neural networks with multiple delays, which have recently been used to construct delay-induced and delay-modulated periodic solutions and their stability in neural mass networks (Coombes and Meijer 2026).

There are also natural links with phase-amplitude and phase-isostable reductions of oscillator networks. Such reductions provide a complementary route to understanding how delayed interactions shape dynamics near and away from a limit cycle, including off-cycle perturbations that are important for transitions between network states. Recent work on phase and amplitude responses for delay equations using harmonic balance (Nicks et al. 2024a), and on oscillator network dynamics using a phase-isostable framework (Nicks et al. 2024b), provides a natural basis for extending the present ideas beyond the Lighthouse setting. One could then compare event-time descriptions of spiking networks with phase-amplitude reductions of more biophysically detailed oscillators and neural mass models.

A further goal is to use adaptive conduction delays to sculpt network attractors. In symmetric networks, delay tuning may be used to organise heteroclinic switching between unstable phase-locked states. In heterogeneous or connectome-like networks, where exact symmetry is absent, a more realistic target may be an excitable network attractor in which states are stable but input-dependent transitions occur between them. The framework developed here provides a starting point for both possibilities: it identifies phase-locked states, determines their stability, and shows how slow delay adaptation can move a system through the corresponding bifurcation structure. This opens the door to studying sequential memory, robust switching, and input-driven computation in networks whose timing architecture is plastic (Ashwin et al. 2024; Rabinovich et al. 2006; Maass et al. 2002).

Finally, the same ideas may be useful for studying robustness and degradation. Since demyelination, ageing and disease can alter conduction speeds, a delay-based theory of phase locking offers a natural language for describing how white matter damage may destabilise coherent activity or alter transitions between network states. This may be relevant for neurodegenerative and demyelinating diseases, such as multiple sclerosis, where altered conduction timing is expected to disrupt coordinated neural communication (Sorrentino et al. 2022). Conversely, adaptive myelination may provide a compensatory mechanism for restoring useful timing relationships after perturbation. Taken together, the results support the view that white matter should be treated not merely as wiring, but as an adaptive dynamical substrate capable of shaping the timing, stability and transitions of spiking network states.

## Data Availability

No datasets were generated or analysed during the current study.

## Appendix A

In this appendix we consider intersections of commensurate asymmetric branches with symmetric branches in the reciprocal 2-node network. Here, we show that if an asymmetric branch attaches smoothly to a symmetric branch in the reciprocal 2-node network of Sect. 5, then the point of attachment is necessarily a stationary point of the symmetric branch in the (τ,T) plane.

For the reciprocal 2-node network, the phase-locked states are determined by the pair of equations

A1 $$\begin{aligned} \mathcal {H}(T,\pm \phi ;\tau )=0, \end{aligned}$$

where, from (26),

A2 $$\begin{aligned} \mathcal {H}(T,\phi ;\tau )=\int _0^T \textrm{d}t \, S\left( P(t)+wP(t+\phi T-\tau )\right) -2\pi . \end{aligned}$$

Here we have written the dependence on τ explicitly. A synchronous branch is defined by

A3 $$\begin{aligned} \mathcal {H}(T,0;\tau )=0, \end{aligned}$$

while an anti-synchronous branch is defined by $\mathcal {H}\left( T,\frac{1}{2};\tau \right) =0$.

We first consider the case of synchrony. Suppose that a smooth asymmetric branch intersects the synchronous branch at a point $(T_*,\tau _*,\phi _*)$ with $\phi _*=0$. Along the asymmetric branch we have

A4 $$\begin{aligned} \mathcal {H}(T,\phi ;\tau )=0, \qquad \mathcal {H}(T,-\phi ;\tau )=0. \end{aligned}$$

Subtracting these two equations gives $\mathcal {H}(T,\phi ;\tau )-$ $\mathcal {H}(T,-\phi ;\tau )=0$. Using a Taylor expansion around ϕ=0 as $\mathcal {H}(T,\phi ;\tau ) = \mathcal {H}(T,0;\tau ) + \phi \left. \partial {\mathcal {H}} /\partial \phi \right| _{(T,0;\tau )} + O(\phi ^2)$ yields $\mathcal {H}(T,\phi ;\tau )-\mathcal {H}(T,-\phi ;\tau ) = 2 \phi \left. \partial {\mathcal {H}} /\partial \phi \right| _{(T,0;\tau )} + O(\phi ^3)$. Dividing by 2ϕ and taking the limit $\phi \rightarrow 0$ yields

A5 $$\begin{aligned} \frac{\partial \mathcal {H}}{\partial \phi }(T_*,0;\tau _*)=0. \end{aligned}$$

To relate this to the slope of the synchronous branch, we differentiate (A3) with respect to τ along a smooth solution T=T(τ). This gives

A6 $$\begin{aligned} \frac{\partial \mathcal {H}}{\partial T}(T,0;\tau ) \frac{\textrm{d}T}{\textrm{d}\tau }+\frac{\partial \mathcal {H}}{\partial \tau }(T,0;\tau )=0. \end{aligned}$$

Hence, provided that

A7 $$\begin{aligned} \frac{\partial \mathcal {H}}{\partial T}(T_*,0;\tau _*)\ne 0, \end{aligned}$$

we obtain

A8 $$\begin{aligned} \frac{\textrm{d}T}{\textrm{d}\tau }(\tau _*)=-\frac{\frac{\partial \mathcal {H}}{\partial \tau }(T_*,0;\tau _*)}{\frac{\partial \mathcal {H}}{\partial T}(T_*,0;\tau _*)}. \end{aligned}$$

Thus it remains to show that $\partial \mathcal {H} /\partial {\tau }=0$ at the point of intersection. This follows directly from the form of $\mathcal {H}$ in (A2). Introducing $A=\phi T-\tau$, we see that ϕ and τ enter $\mathcal {H}$ only through the combination *A*. Differentiating (A2) directly with respect to τ and ϕ shows that

A9 $$\begin{aligned} \frac{\partial \mathcal {H}}{\partial \tau }(T,\phi ;\tau )=-\frac{1}{T}\frac{\partial \mathcal {H}}{\partial \phi }(T,\phi ;\tau ). \end{aligned}$$

Evaluating (A9) at the point of attachment and using (A5) we obtain

A10 $$\begin{aligned} \frac{\partial \mathcal {H}}{\partial \tau }(T_*,0;\tau _*)=0. \end{aligned}$$

Substitution into (A6) now gives

A11 $$\begin{aligned} \frac{\textrm{d}T}{\textrm{d}\tau }(\tau _*)=0. \end{aligned}$$

Hence the synchronous branch is stationary at the point where the asymmetric branch attaches. The proof for the anti-synchronous branch is identical and we do not present it here. We have therefore established the following: *if an asymmetric branch attaches smoothly to either the synchronous branch* ϕ=0 *or the anti-synchronous branch* ϕ=1/2 *, then the point of attachment is a stationary point of the corresponding symmetric branch in the* (τ,T) *plane, provided that* $\partial {\mathcal {H}} / \partial {T}\ne 0$ *at that point.*
